# Divergent Role of ULK1 to Balance Mitochondrial Homeostasis and Bioenergetics in Ovarian Cancer Spheroids

**DOI:** 10.3390/cancers18111746

**Published:** 2026-05-27

**Authors:** Jack D. Webb, Matthew J. Borrelli, Yudith Ramos Valdés, Trevor G. Shepherd

**Affiliations:** 1 The Mary & John Knight Translational Ovarian Cancer Research Unit, Verspeeten Family Cancer Centre, London, ON N6A 5W9, Canada; jwebb47@uwo.ca (J.D.W.);; 2 Department of Anatomy & Cell Biology, Schulich School of Medicine & Dentistry, Western University, London, ON N6A 5C1, Canada; 3 Department of Oncology, Schulich School of Medicine & Dentistry, Western University, London, ON N6A 5C1, Canada; 4 Department of Obstetrics & Gynaecology, Schulich School of Medicine & Dentistry, Western University, London, ON N6A 5C1, Canada

**Keywords:** ULK1, autophagy, ovarian cancer, spheroids, mitochondria, oxidative phosphorylation

## Abstract

Background/objectives: Epithelial ovarian cancer (EOC) is the deadliest gynaecologic malignancy, largely due to late-stage diagnosis and ineffective therapy. EOC commonly spreads through the peritoneal cavity as multicellular spheroids, which are metastatic structures that enhance survival under detachment stress, promote dissemination, and contribute to therapeutic resistance. We previously showed that ULK1, a serine/threonine kinase classically linked to macroautophagy initiation, supports EOC progression, suggesting non-canonical roles in spheroid biology and pathogenesis. Methods: CRISPR/Cas9 *ULK1* knockout (*ULK1*KO) models were generated in OVCAR8, HEYA8, and ES2 cells. Mitochondrial degradation phenotypes were assessed in spheroids by immunoblotting and fluorescence microscopy. Label-free proteomics with bioinformatic pathway analysis identified ULK1-associated programs in EOC spheroids. Bioenergetic consequences were quantified using Seahorse ATP-Rate assays. Therapeutic interactions were evaluated using multi-dose combination matrices testing the ULK1 inhibitor DCC-3116 with metformin. Results: ULK1 modulated mitochondrial degradation in a cell-line-specific manner, either promoting or protecting against mitochondrial loss through mechanisms that were uncoupled from canonical autophagy machinery. Proteomic and bioinformatic analyses revealed significant alterations in mitochondria-related processes, aligning with emerging ULK1 functions in mitochondrial homeostasis. *ULK1* loss broadly reduced OXPHOS complex proteins in EOC spheroids and consistently decreased hexokinase 2 (HK2), indicating coordinated metabolic remodeling. Seahorse profiling mirrored these shifts: OVCAR8 *ULK1*KO spheroids showed reduced OCR and ATP production, whereas HEYA8 and ES2 *ULK1*KO spheroids exhibited increased mitochondrial ATP production. Combination matrices showed potential synergy between DCC-3116 and metformin. Conclusions: These data show that ULK1 differentially regulates mitochondrial degradation across EOC spheroid models through potential mechanisms alternative to canonical autophagy machinery, while reshaping spheroid metabolism and revealing potential therapeutic vulnerabilities in advanced EOC.

## Simple Summary

Epithelial ovarian cancer (EOC) is the deadliest cancer of the female reproductive system because it is often diagnosed late and remains difficult to treat after it has spread. EOC cells commonly disseminate as multicellular clusters called spheroids, which promote survival in suspension, enhance resistance to stress and therapy, and support the formation of new tumors. Cells within spheroids rely on autophagy, a conserved survival pathway regulated by unc-51-like autophagy activating kinase 1 (ULK1). We previously showed that ULK1 contributes to EOC progression, with additional results suggesting it has important functions beyond the canonical role in autophagy initiation. Here, we investigated ULK1 function impacting mitochondrial homeostasis and metabolism in EOC spheroids. We found that ULK1 regulates mitochondrial degradation through mechanisms that may be uncoupled from canonical autophagy, while *ULK1* loss remodels spheroid energy metabolism. Finally, combined inhibition of ULK1 using DCC-3116 and oxidative phosphorylation using metformin showed potential enhanced therapeutic efficacy, revealing new opportunities to target advanced EOC.

## 1. Introduction

Epithelial ovarian cancer (EOC) ranks as the most lethal gynaecological cancer in developed countries, primarily due to its late-stage diagnosis and the ineffectiveness of treatments for chemo-resistant disease [1]. Standard treatment typically involves cytoreductive surgery and platinum–taxane combination chemotherapy, with maintenance approaches such as PARP inhibitors and/or bevacizumab used in selected patients. Despite these advances, many patients relapse, highlighting the need to identify stress-adaptive pathways that support EOC persistence and therapeutic resistance [2]. EOC typically spreads by the direct dissemination of tumor cells into the peritoneal cavity [2,3], where they aggregate to form spheroids [4]. These spheroids are central to the metastatic process, as they exhibit increased adhesion and invasion abilities, and resist cytotoxic chemotherapy [5,6]. In addition, spheroids undergo various phenotypic changes, such as the induction of autophagy [7,8]. Autophagy is a well-preserved and tightly controlled metabolic degradation process, where proteins and organelles are broken down in the lysosome [9,10]. This process releases lysosome-derived metabolic by-products, including amino acids and other molecules, which provide nutrients and energy to support essential cellular functions during nutrient scarcity or metabolic stress [11,12]. Autophagy occurs at low basal levels to maintain cellular homeostasis but is rapidly upregulated in response to stress [13]. The serine/threonine kinase ULK1 (Unc-51-like kinase 1) is recognized as the primary regulator of autophagy, responding to upstream signals about nutrient and energy availability to trigger the autophagic process. In nutrient-abundant conditions, Mechanistic Target of Rapamycin Complex 1 (mTORC1) phosphorylates ULK1, thus restraining its activity and autophagy initiation. Conversely, the absence of nutrients leads to mTORC1 deactivation and ULK1 dephosphorylation, while AMPK (AMP-activated protein kinase) phosphorylates and activates ULK1 [9]. Consequently, ULK1 and autophagy are essential in maintaining cellular equilibrium by eliminating impaired proteins and organelles and preserving intracellular energy supplies to meet the demands of the cell [13]. Because mitochondria both fuel cellular energetics and generate damaging ROS (reactive oxygen species), ULK1’s control of autophagy and related pathways can directly determine mitochondrial homeostasis.

To maintain mitochondrial homeostasis and energy metabolism, cells have developed several quality control mechanisms, including catabolic pathways such as mitophagy, but also mitochondrial biogenesis [14]. Mitophagy, a specific type of macroautophagy, is one such mechanism that selectively eliminates dysfunctional or damaged mitochondria [15,16]. Mitochondria adapt to meet cellular energy and physiological demands through continuous fission and fusion processes [17]. Increased respiratory activity and ROS production lead to mitochondrial damage, triggering mitophagy [18,19]. Indeed, ULK1 is upregulated in response to mitophagy, translocating to fragmented mitochondria through receptor-mediated interactions between autophagic machinery and mitochondria [20,21,22,23], while it can also balance cellular reliance on glycolysis and mitochondrial metabolism [24,25]. Mitochondria play a critical role in cellular metabolism and overall physiological function, with mitochondrial damage linked to a wide range of diseases. Dysfunctions in mitochondrial degradation can disturb cellular balance, potentially leading to tumor development, illustrating the intricate relationship between mitochondria, autophagy, and the onset and progression of cancer. Mitochondria are pivotal in cancer, contributing significantly to the invasiveness and metastatic traits of tumors [15].

We have previously identified that ULK1 is required for autophagy initiation and mediates metastatic progression in EOC, contributing to spheroid invasion, survival, and tumour growth, highlighting its canonical and non-canonical functions [8]. Additionally, label-free proteomic mass spectrometry and bioinformatic analysis revealed significant alterations in pathways and processes related to mitochondria, including oxidative phosphorylation (OXPHOS), electron transport chain, and mitochondrial gene expression. This aligns with recent studies that have uncovered new ULK1 functions in mitochondrial homeostasis [22,26,27,28]. We have previously observed that EOC spheroids display varied patterns in the regulation of mitochondrial dynamics, including fission, fusion, and mitophagy [29], and have increased OXPHOS protein expression relative to adherent cells [30]. However, few studies have investigated ULK1-mediated mitochondrial homeostasis in EOC spheroids. Building on our prior work investigating the role of ULK1 in EOC progression, we aimed to elucidate non-canonical functions of ULK1 that may contribute to EOC metastasis and uncover additional therapeutic vulnerabilities in the absence of ULK1.

## 2. Materials and Methods

### 2.1. Antibodies and Reagents

Antibodies against ULK1 (#8054S), LC3B (#2775S), Beclin1 S30 (#5410S), Beclin1 (#3738S), ATG5 (#2630S), ATG7 (#8558S), VDAC (#4662S), PINK1 (#6946S), and the Glycolysis Antibody Sampler Kit (#8337) were purchased from Cell Signaling Technology (Danvers, MA, USA). Total OXPHOS Rodent WB Antibody Cocktail (ab410113) and mCherry (ab167453; 1:500) were purchased from Abcam (Cambridge, UK). Anti-actin antibody (A2066; 1:25,000) was purchased from Millipore (Burlington, MA, USA). Antibodies against tubulin (T5168; 1:40,000) and vinculin (V9264; 1:25,000) were purchased from Sigma-Aldrich (Burlington, MA, USA). Horseradish peroxidase (HRP)-conjugated antibodies against mouse IgG (NA931; 1:10,000) and rabbit IgG (NA934; 1:10,000) were purchased from Cytiva (Malborough, MA, USA). Antibodies were diluted in tris-buffered saline-Tween 20 containing either 5% bovine serum albumin or non-fat milk 1:1000. Brefeldin A (#9972) and Chloroquine (#14774) were purchased from Cell Signaling Technology (Danvers, MA, USA). DCC-3116 (#HY-160699) and metformin (#HY-17471A) were purchased from MedChemExpress (Monmouth Junction, NJ, USA).

### 2.2. Generation of ULK1KO Cell Lines

Generation of OVCAR8-*ULK1*KO, HEYA8-*ULK1*KO, and ES2-*ULK1*KO cell lines has been previously described [8].

### 2.3. Cultured Cell Lines

The cell lines OVCAR8, OVCAR8-*ULK1*KO, HEYA8, and HEYA8-*ULK1*KO were grown in RPMI-1640 (#350-700 CL, Wisent (Saint-Jean-Baptiste, QC, Canada)), whereas ES2 and ES2-*ULK1*KO were grown in DMEM/F12 medium (#11320033, Thermo Fisher Scientific, Waltham, MA, USA). All growth media were supplemented with 10% fetal bovine serum. OVCAR8, HEYA8, and ES2 cells were procured from the American Type Culture Collection (ATCC; Manassas, VA, USA). Adherent cells were sustained on tissue culture-treated polystyrene (Sarstedt, Newton, NC, USA), and spheroids were maintained in Ultra-Low Attachment (ULA) cluster plates (Corning, NY, USA). All cell lines were authenticated through short tandem repeat analysis by the Center for Applied Genomics (The Hospital for Sick Children, Toronto, ON, Canada) and routinely examined for mycoplasma using a Universal Mycoplasma Detection Kit (30-1012K; ATCC; Manassas, VA, USA).

### 2.4. Plasmids

Parental and *ULK1*KO cells were transfected with either the mCherry-eGFP-LC3B autophagy or mCherry-eGFP-FIS1_101–152_ mitophagy-reporter plasmid. pBABE-puro mCherry-eGFP-LC3B was a gift from Jayanta Debnath (Addgene plasmid #22418; http://n2t.net/addgene:22418 (accessed on 10 March 2022); RRID:Addgene_22418) [31], while pBABE-puro.mCherry-eGFP-FIS1_101–152_ was obtained from the MRC PPU Reagents and Services facility (DU40799; University of Dundee). The mCherry-eGFP-LC3B cDNA was placed under control of a CMV promoter, as described in [29]. Briefly, mCherry-eGFP-LC3B cDNA was cloned into pcDNA3 to enable G-418 selection and CMV promoter-driven expression. Using the Q5 Site-Directed Mutagenesis Kit (New England Biolabs, E0554; Ipswich, MA, USA), the unique NgoMIV and SalI sites in pBABE-puro.mCherry-eGFP-LC3B were converted to HindIII and BamHI sites, respectively. Following confirmation by diagnostic digest, mCherry-eGFP-LC3B was excised with HindIII/BamHI and ligated into similarly digested pcDNA3 to generate pcDNA3.mCherry-eGFP-LC3B. Gel extractions were performed using the QIAEX II Gel Extraction Kit (Qiagen, 20021; Germantown, MA, USA) and ligations were performed using T4 DNA Ligase (New England Biolabs, M0202; Ipswich, MA, USA) according to the manufacturers’ protocols. After transfection, cells were selected in complete medium containing G-418 (400 μg/mL) for two weeks, cultured without G-418 for four additional weeks, and then sorted by FACS to isolate double-positive cells.

### 2.5. Preparation of Whole-Cell Lysates

For whole-cell protein analysis, adherent cells were seeded at 0.75–1 × 10^6^ cells in 10 mL medium in 10 cm dishes, while spheroid cells were seeded at 1–3 × 10^6^ cells in 15 mL medium in 35 mm ULA wells. Seeding densities were optimized to ensure sufficient protein yield for each cell line.

Adherent cells were washed twice with cold PBS and scraped into modified RIPA buffer containing 50 mM HEPES pH 7.4, 150 mM NaCl, 10% glycerol, 1.5 mM MgCl_2_, 1 mM EGTA, 1% Triton X-100, 0.1% SDS, 1 mM Na_3_VO_4_, 10 mM NaF, 1 mM PMSF, 1× SIGMAFAST protease inhibitor cocktail, and 10 mM beta-glycerophosphate. Spheroids were collected by centrifugation at 800× *g* for 4 min at 4 °C, washed twice with cold PBS, and lysed in modified RIPA buffer. Lysates were vortexed, subjected to one freeze–thaw cycle, and clarified by centrifugation at maximum speed for 20 min at 4 °C.

### 2.6. Immunoblot Analysis

Immunoblotting was performed using the Bio-Rad Mini-PROTEAN II Electrophoresis System according to the manufacturer’s guidelines, with in-house prepared gels made from 30% acrylamide/bis solution 37.5:1 (Bio-Rad, 1610158; Hercules, CA, USA). Densitometric analysis was performed using Image Lab 6.05 software.

### 2.7. SPoRTS Analysis

Time-course fluorescence images of mitoR and autoR spheroids were captured in the RFP and eGFP channels using an IncuCyte S3 live-cell analysis system, with exposure times of 400 ms and 300 ms, respectively. Spatial analyses of the RFP:eGFP ratio were performed using the profile method from the original SPoRTS workflow [32]. Default settings for SPoRTS were used for all analyses, except for “rmaps_compare_conditions” setting, which was set to “True”.

### 2.8. Transient Knockdown of ULK1, ATG5, and ATG7

Cells were seeded in 6-well plates (1.5  ×  105 cells/well) and transfected using DharmaFECT1 (1/500 final dilution) as per the manufacturer’s protocol (Dharmacon, *Cat# T-2001*; Lafayette, CO, USA) using a final total siRNA concentration of 10 nM. ULK1 (L-005049-00-0005), ATG5 (M-004374-04), and ATG7 (M-020112-01) ON-TARGETplus SMARTpool or ON-TARGETplus Non-targeting Pool (D-001810-10-20) were used (Dharmacon; Lafayette, CO, USA). Medium was aspirated 24 h after transfection, and 5 mL of fresh medium was added to each well. Cells were trypsinized, counted and seeded 48 h later for experiments.

### 2.9. MitoSOX

Cells were seeded in a 96-well round-bottom ULA plate at a density of 2000 cells per well in 100 μL of medium. After 72-h, cells were incubated with MitoSOX (#M36008, Thermo Fischer, Waltham, MA, USA) with a final dilution of 5 uM. Fluorescent images were captured in the IncuCyte S3 Live-Cell Analysis System (Sartorius, Oakville, ON, Canada). Analyses of whole-spheroid red fluorescence intensity were conducted using the intensity method described in the original version of SPoRTS [32].

### 2.10. Proteomic Mass Spectrometry

Protein extraction and mass spectrometry analysis were performed on OVCAR8 wild-type and OVCAR8-*ULK1*KO 24-h spheroids as previously described [8]. Pathway analysis was performed using gene set enrichment analysis (GSEA) [33], and KEGG [34] (http://bioinformatics.sdstate.edu/go/ (accessed on 18 February 2024)) and Reactome databases [35] (https://reactome.org (accessed on 18 February 2024)).

### 2.11. Seahorse XF Real-Time ATP Rate Assay

Agilent Seahorse XF Real-Time ATP Rate Assay (#103592-100, Santa Clara, CA, USA) was performed in the Seahorse XFe24 analyzer (#102238-100) according to the manufacturer’s protocols. Adherent cells were seeded at 200,000 cells/mL on Seahorse 24-well cell culture plates and allowed to grow for 24 h; spheroids were grown in 96-well ULA plates for 48 h, then 15 spheroids were combined per well of a Seahorse 24-well cell culture plate. Spheroid washing and data normalization were adapted from [36]. Cell number was estimated for adherent cells using N_t_ = N_0_2^t/Td^, where N0 = 50,000 cells, from 250 μL of cells at 200,000 cells/mL, and T_d_ = 48.85 h. Doubling time (T_d_) was averaged from previous studies [37,38].

### 2.12. Synergy Finder Analysis

Cells were seeded in 96-well round-bottom ULA plates at 2000 cells/well in 100 μL medium. Twenty-four hours after seeding, spheroids were treated with 96 combinations of DCC-3116 and metformin for 72 h. Cell viability was assessed by incubating spheroids with a 1:1 dilution of CellTiter-Glo (Promega, #G7572; Madison, WI, USA) for 60 min in the dark on a plate rocker. Well contents were then transferred to 96-well opaque white plates, and luminescence was measured using an Agilent BioTek Synergy H1 plate reader (Agilent Technologies, Mississauga, ON, Canada). Normalized data were formatted according to the SynergyFinder user manual (https://synergyfinder.aittokallio.group/synfin_docs/ (accessed on 3 December 2025)) and analyzed on the SynergyFinder platform using the ZIP Synergy Score model [39,40].

### 2.13. Statistical Analysis

Statistical analyses were performed using GraphPad Prism 10 (GraphPad Software) and the details for specific statistical tests are described in each figure legend.

## 3. Results

### 3.1. ULK1 Deficiency Impacts Mitochondrial Degradation

**Figure 1:**
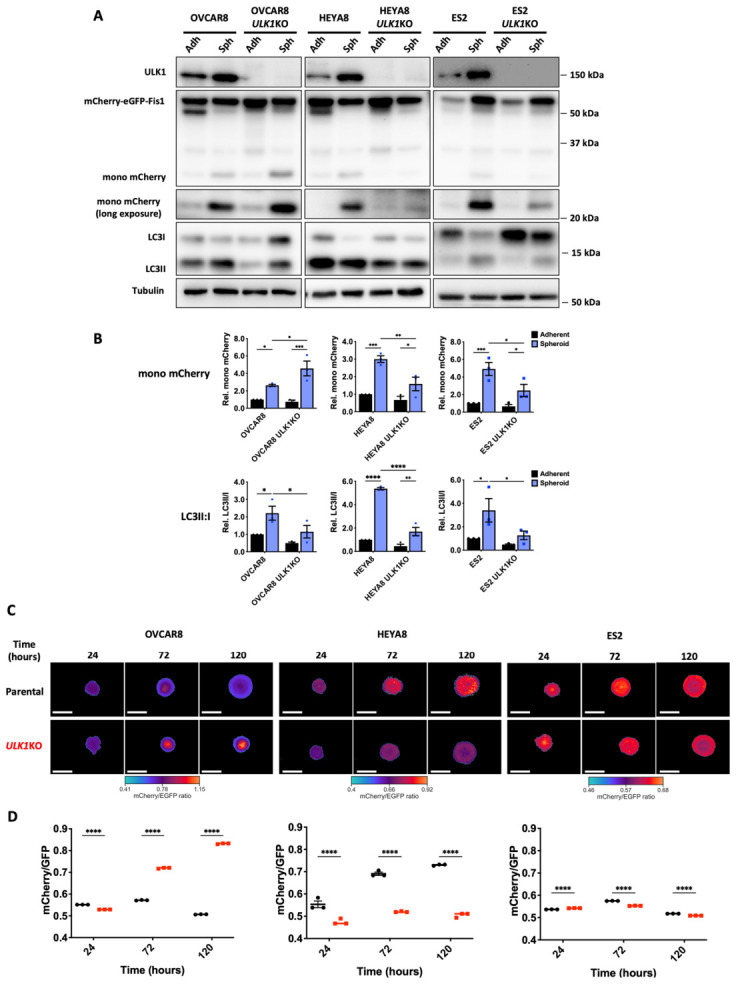
*ULK1* deficiency exhibits cell-line-specific impacts on mitochondrial degradation. (**A**) OVCAR8, HEYA8, and ES2 parental and *ULK1*KO cells were seeded in adherent and spheroid culture. Protein lysates were harvested 72 h after seeding for Western blot analysis of mono-mCherry and LC3. (**B**) Densitometric analysis of mono-mCherry (mono-mCherry/(mono-mCherry + mCherry-eGFP-FIS1_101–152_)) and LC3II:I relative to expression in parental adherent conditions (*N* = 3). Data displayed as mean ± SEM; two-way ANOVA followed by Šidák’s multiple comparisons test, * *p* < 0.05, ** *p* < 0.01, *** *p* < 0.001, **** *p* < 0.0001. (**C**) Representative heatmaps generated with SPoRTS (Spatial Profiling of Ratiometric Trends in Spheroids) from paired mCherry and eGFP images of OVCAR8, HEYA8, and ES2 parental and *ULK1*KO-mitoR spheroids. Per-pixel mCherry/eGFP ratios are displayed on a common color scale (warm colors = higher ratio), providing spatial visualization of mitochondrial delivery to acidic compartments. Images were processed with identical analysis settings; color bars indicate the ratio range (*N* = 3, with at least 6 technical replicates per biological replicate). Scale bar, 400 µm. (**D**) Quantification of reporter activity as mCherry/eGFP ratio over time (24–168 h in suspension) across the entire spheroid. Black and red symbols reflect parental and *ULK1*KO spheroids, respectively. Data displayed as mean ± SEM (*N* = 3, with at least 8 technical replicates per biological replicate); multiple Student’s *t*-test, **** *p* < 0.0001. Original western blots are presented in File S1.

Beyond its well-characterized role in initiating macroautophagy under nutrient deprivation, ULK1 is a critical regulator of selective autophagy, facilitating the targeted clearance of damaged or excess organelles, including mitochondria via mitophagy. We have previously observed spatial and temporal differences in autophagy and mitophagy in EOC spheroids [29]; thus, we sought to assess the role of ULK1 in regulating mitophagy in this context. To assess mitophagy in EOC spheroids, we used a mitochondria-targeted mCherry-eGFP-FIS1_101–152_ reporter [41]. In this system, eGFP is quenched in acidic autolysosomes while mCherry remains stable, allowing detection of mitochondrial delivery to these degradative compartments. Molecular analysis via immunoblotting demonstrated increased monomeric mCherry and the LC3II:I ratio in parental EOC cell line spheroids, consistent with both autophagy and mitophagy activation. By contrast, *ULK1* loss produced cell line-specific effects: OVCAR8 *ULK1*KO spheroids showed a further rise in mono-mCherry, whereas HEYA8 *ULK1*KO and ES2 *ULK1*KO spheroids displayed significant decreases in mono-mCherry, even though ULK1-deficient lines were autophagy-impaired (Figure 1A,B). Live-cell fluorescence imaging revealed an elevated mCherry/eGFP ratio in OVCAR8 *ULK1*KO spheroids, yet a reduced ratio in HEYA8 and ES2 *ULK1*KO spheroids beyond 72 h (Figure 1C,D). Together, these data indicate that ULK1 may differentially control mitochondrial degradation among these EOC cell line models; intriguingly, mitochondrial clearance may proceed despite impaired canonical autophagy, pointing to potential ULK1-dependent, autophagy-independent mitochondrial degradation mechanisms.

### 3.2. ULK1 Differentially Regulates Mitochondrial Degradation Through Mechanisms Uncoupled from ATG5/7-Dependent Autophagic Flux

**Figure 2:**
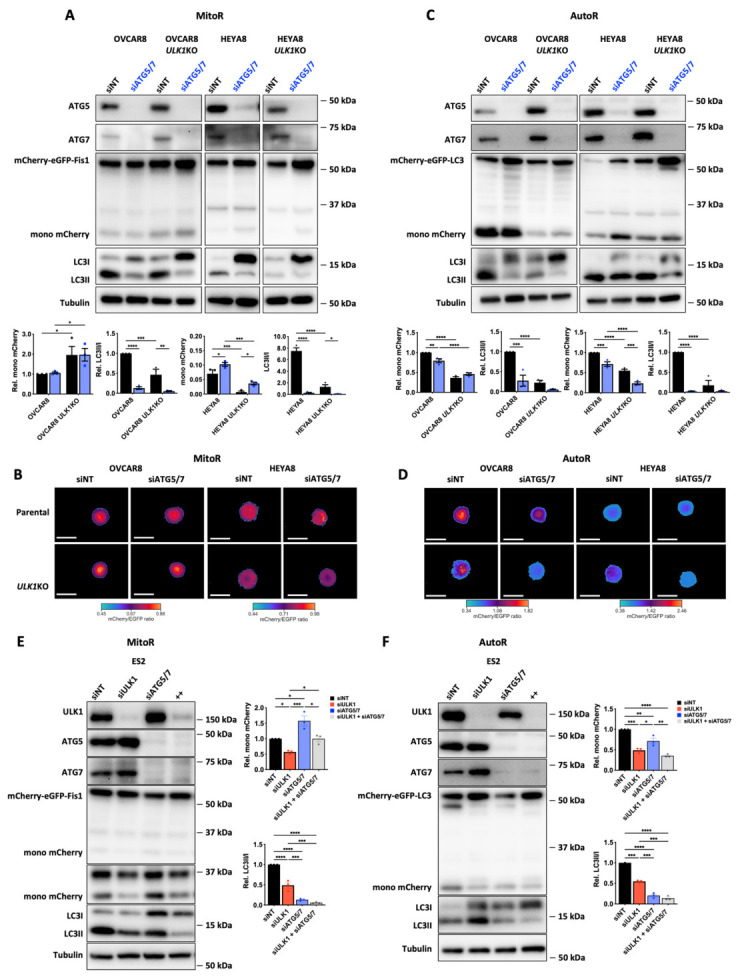
ULK1 regulates mitochondrial degradation independently of ATG5/7-mediated autophagy. Western blot analysis of OVCAR8 and HEYA8 (parental and *ULK1*KO) (**A**) mitophagy-reporter and (**C**) autophagy-reporter cells transfected with non-targeting siRNA (siNT), siATG5/7, and siULK1. Cells were transfected in adherent conditions for 48 h before trypsinizing, counting, and seeding in spheroid culture. Protein lysates were collected 72 h after seeding in spheroid culture. Densitometric analysis of mono-mCherry (mono-mCherry/(mono-mCherry + mCherry-eGFP-FIS1)) and LC3II:I relative to expression in siNT conditions (*N* = 3). Black and blue symbols reflect siNT and siATG5/7 conditions, respectively. Data displayed as mean ± SEM; two-way ANOVA followed by Šidák’s multiple comparisons test, * *p* < 0.05, ** *p* < 0.01, *** *p* < 0.001, **** *p* < 0.0001. Representative heatmaps generated with SPoRTS (Spatial Profiling of Ratiometric Trends in Spheroids) from paired mCherry and eGFP images of (**B**) mitophagy-reporter and (**D**) autophagy-reporter spheroids under siNT or siATG5/7 conditions. Per-pixel mCherry/eGFP ratios are displayed on a common color scale (warm colors = higher ratio), providing spatial visualization of mitochondrial delivery to acidic compartments. Images were processed with identical analysis settings; color bars indicate the ratio range (*N* = 3, with at least 6 technical replicates per biological replicate). Scale bar, 400 µm. Western blot analysis of ES2 (**E**) mitophagy-reporter and (**F**) autophagy-reporter cells transfected with non-targeting siRNA (siNT), siATG5/7, siULK1, or the combination (siULK1 + siATG5/7) as indicated. Cells were transfected in adherent conditions for 48 h before trypsinizing, counting, and seeding in spheroid culture. Protein lysates were collected 72 h after seeding in spheroid culture. Densitometric analysis of mono-mCherry (mono-mCherry/(mono-mCherry + mCherry-eGFP-FIS1)) and LC3II:I relative to expression in siNT conditions (*N* = 3). Data displayed as mean ± SEM; two-way ANOVA followed by Šidák’s multiple comparisons test, * *p* < 0.05, ** *p* < 0.01, *** *p* < 0.001, **** *p* < 0.0001. Original western blots are presented in File S1.

To test whether ULK1-associated changes in mitochondrial degradation depend on core autophagy machinery, we further utilized our cells expressing mCherry-eGFP-FIS1_101–152_ by performing knockdown of *ATG5* and *ATG7* in parental and *ULK1*KO cells. In OVCAR8 *ULK1*KO spheroids, the increase in mono-mCherry persisted despite *ATG5/7* knockdown, suggesting that ULK1 normally restrains mitochondrial degradation in this model, while the enhanced degradation caused by *ULK1* loss can occur independently of ATG5/7-mediated canonical autophagy (Figure 2A). In contrast, *ULK1* loss in HEYA8 and ES2 spheroids decreased mitochondrial degradation as seen with mono-mCherry levels, indicating that ULK1 supports mitochondrial turnover in these models. However, *ATG5/7* knockdown paradoxically enhanced mitochondrial degradation, suggesting that canonical autophagy may act to preserve mitochondria in this context. Among all cell lines, si*ATG5/7* significantly decreased LC3II:I alone and with *ULK1* loss, confirming impaired autophagy activation. Live-cell imaging of parental and *ULK1*KO-mitoR spheroids deficient for *ATG5* and *ATG7* phenocopied these molecular readouts (Figure 2B). To confirm that macroautophagy was being affected, we repeated key perturbations in matched autophagy reporter lines (mCherry-eGFP-LC3B) by immunoblot. As expected, *ULK1*KO and si*ATG5/7* both decreased mono-mCherry and LC3II:I in EOC cell lines (Figure 2C,D), confirming that our genetic tools directly impair autophagic flux. In contrast, these same genetic perturbations exerted divergent effects on mitochondrial degradation as indicated using analogous dual fluorescence mitophagy-reporter cell lines, indicating that ULK1 can differentially modulate mitochondrial degradation through mechanisms that may be uncoupled from ATG5/7-dependent canonical autophagy.

To this point, these experiments have utilized EOC cell lines with stable genetic ablation of ULK1. To determine whether *ULK1* loss affects mitochondrial degradation through acute changes rather than long-term adaptation, we performed transient *ULK1* knockdown in ES2 mitophagy-reporter-expressing spheroids. Transient *ULK1* knockdown significantly reduced mono-mCherry levels, phenocopying our *ULK1*KO data. Interestingly, *ATG5/7* knockdown increased mono-mCherry levels as we observed already (Figure 2A,B), suggesting enhanced mitochondrial degradation, while combined siULK1 + siATG5/7 attenuated this effect. As expected, knockdown of either *ULK1* or *ATG5/7* or in combination significantly decreased LC3II:I, confirming impaired autophagic flux (Figure 2E). Importantly, repeating these knockdowns in matched ES2-autoR lines again showed that si*ULK1* and si*ATG5/7* (alone and combined) decreased mono-mCherry and LC3II:I (Figure 2F), reinforcing the idea that mitochondrial degradation occurs in parallel with autophagy due to ULK1-mediated regulation.

OVCAR8 *ULK1*KO spheroids possessed enhanced mitochondrial degradation that appeared to be autophagy independent. To determine if this degradation was still lysosome-dependent, we treated OVCAR8 mitoR-expressing spheroids with the lysosomotropic agent chloroquine (CQ) for 24 h and performed immunoblot analysis. Treatment with CQ significantly decreased mono-mCherry in parental spheroids, while attenuating the significant increase observed in *ULK1*KO spheroids (Supplementary Figure S1), supporting a lysosome-dependent mechanism for the mitochondrial degradation.

Given that enhanced mitochondrial degradation in OVCAR8 *ULK1*KO spheroids persisted despite *ATG5/7* knockdown yet remained sensitive to CQ, we next asked whether altered vesicular trafficking could contribute to this phenotype. Reanalysis of our previous OVCAR8 *ULK1*KO spheroid proteomics [8] identified enrichment of protein secretion and Golgi-associated vesicle signatures, with ADP-ribosylation factor 1 (ARF1) among the most significantly enriched proteins within the Hallmark protein secretion gene set (Supplementary Figure S2A). Because brefeldin A (BFA) disrupts ARF-GEF/ARF-dependent Golgi and secretory trafficking [42], we treated OVCAR8-mitoR spheroids with BFA. BFA increased mono-mCherry accumulation in parental spheroids, partially phenocopying the enhanced mitochondrial reporter degradation observed in *ULK1*KO spheroids. BFA further increased mono-mCherry levels in *ULK1*KO spheroids, indicating that mitochondrial reporter degradation in this model remains responsive to pharmacological disruption of Golgi/secretory trafficking (Supplementary Figure S2B–D). Chloroquine attenuated the enhanced mono-mCherry signal observed in both *ULK1*KO and BFA-treated spheroids (Supplementary Figure S2E), supporting that this increased mitochondrial reporter processing remains lysosome-associated.

Collectively, these results suggest that ULK1 has the potential to either promote or protect against lysosomal-associated mitochondrial degradation in EOC spheroids, but neither process is affected by ATG5/7-mediated canonical autophagy. These findings support an important role for ULK1 in controlling mitochondrial homeostasis beyond its classical function in macroautophagy initiation.

### 3.3. ULK1-Mediated Mitochondrial Degradation Correlates with ROS Generation

**Figure 3:**
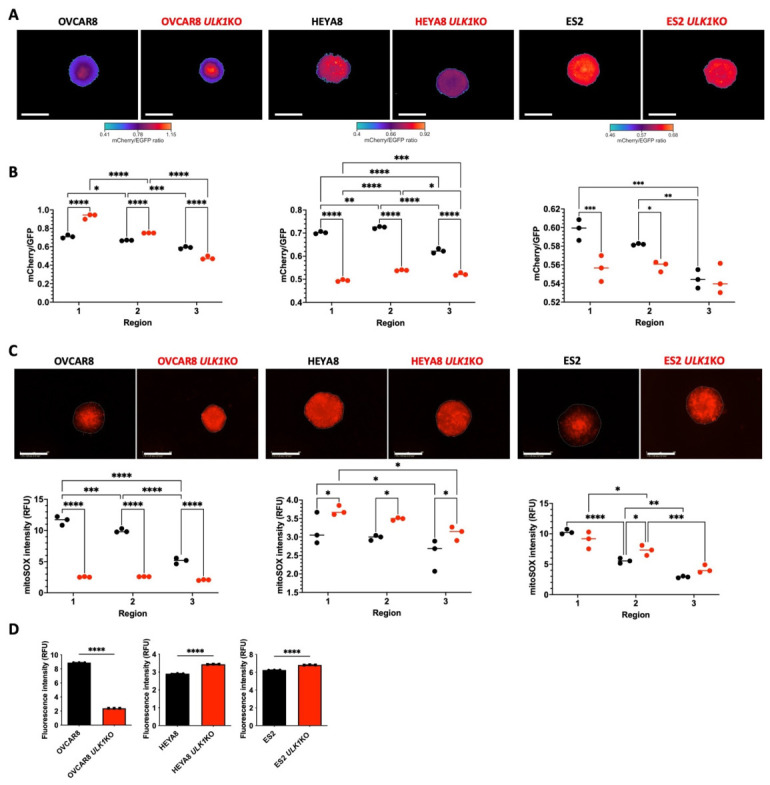
ULK1 and mitochondrial degradation status correlate with mitochondrial ROS. (**A**) Representative heatmaps generated with SPoRTS from paired mCherry and eGFP images of OVCAR8, HEYA8, and ES2 parental and *ULK1*KO mitophagy-reporter spheroids at 72 h (from Figure 1A). Per-pixel mCherry/eGFP ratios are displayed on a common color scale (warm colors = higher ratio), providing spatial visualization of mitochondrial delivery to acidic compartments. Images were processed with identical analysis settings; color bars indicate the ratio. Scale bar, 400 µm. (**B**) Spatial SPoRTS profiles of the mCherry/eGFP ratio plotted as a function of distance from the spheroid core (pixels). Data displayed as mean ± SEM (*N* = 3, with at least 8 technical replicates per biological replicate); two-way ANOVA followed by Šidák’s multiple comparisons test, * *p* < 0.05, ** *p* < 0.01, *** *p* < 0.001, **** *p* < 0.0001. (**C**) Parental and *ULK1*KO spheroids were cultured for 72 h, then incubated with MitoSOX (mitochondrial superoxide) for 12 h. Dotted line reflects spheroid border. Spatial mitoSOX profiles were quantified using SPoRTs and plotted as a function of distance from the spheroid core (pixels). Black and red symbols reflect parental and *ULK1*KO spheroids, respectively. Data reflect fluorescence intensity (RFU) and are displayed as mean ± SEM (*N* = 3, with at least 5 technical replicates per biological replicate). Two-way ANOVA followed by Šidák’s multiple comparisons test, * *p* < 0.05, ** *p* < 0.01, *** *p* < 0.001, **** *p* < 0.0001. Scale bar, 400 µm. (**D**) Average mitoSOX intensity in spheroids at 72 h. Data displayed as mean ± SEM (*N* = 3, with at least 8 technical replicates per biological replicate); Student’s *t*-test, **** *p* < 0.0001.

Given that mitochondria are a major source of reactive oxygen species (ROS), and mitochondrial degradation represents a fundamental homeostatic mechanism, we investigated whether the impact of *ULK1* loss on mitochondrial degradation alters ROS production. Using our recently developed SPoRTS analysis [32] for spatio-temporal monitoring of fluorescence-based reporters, we assessed spatial patterns of mitochondrial degradation in EOC spheroids. In both OVCAR8 parental and *ULK1*KO spheroids, mitochondrial degradation was highest in the spheroid core, with *ULK1*KO spheroids showing significantly increased degradation. Similarly, HEYA8 and ES2 parental spheroids exhibited peak mitochondrial degradation in the core; however, HEYA8 *ULK1*KO spheroids displayed markedly reduced degradation across all spheroid regions, while degradation was reduced in core and intermediate regions in ES2 *ULK1*KO spheroids (Figure 3A,B).

We next examined whether changes in mitochondrial degradation corresponded with alterations in mitochondrial ROS levels using mitoSOX fluorescence measurements. The spatial distribution of mitochondrial ROS showed partial correspondence with mitochondrial degradation patterns among cell line models. OVCAR8 parental and *ULK1*KO spheroids maintained similar mitochondrial ROS spatial distributions, with elevated levels concentrated in the core relative to the periphery. However, OVCAR8 *ULK1*KO spheroids exhibited significantly reduced mitochondrial ROS levels across all regions compared to parental spheroid controls. In contrast, both HEYA8 and ES2 parental spheroids demonstrated elevated mitochondrial ROS in core regions, but with distinct ULK1-dependent alterations. HEYA8 *ULK1*KO spheroids showed elevated mitochondrial ROS levels throughout the entire spheroid, while ES2 *ULK1*KO spheroids exhibited increased mitochondrial ROS specifically in intermediate regions between the core and periphery (Figure 3C). Overall, OVCAR8 *ULK1*KO spheroids exhibited significantly lower global mitochondrial ROS than parental cells, whereas HEYA8 and ES2 *ULK1*KO spheroids showed significantly higher global mitochondrial ROS relative to their respective parental counterparts (Figure 3D). Together, these data show that ULK1-dependent changes in mitochondrial degradation are accompanied by corresponding alterations in mitochondrial ROS, supporting an association between mitochondrial turnover and ROS status.

### 3.4. ULK1 Regulation of Energy Metabolism

**Figure 4:**
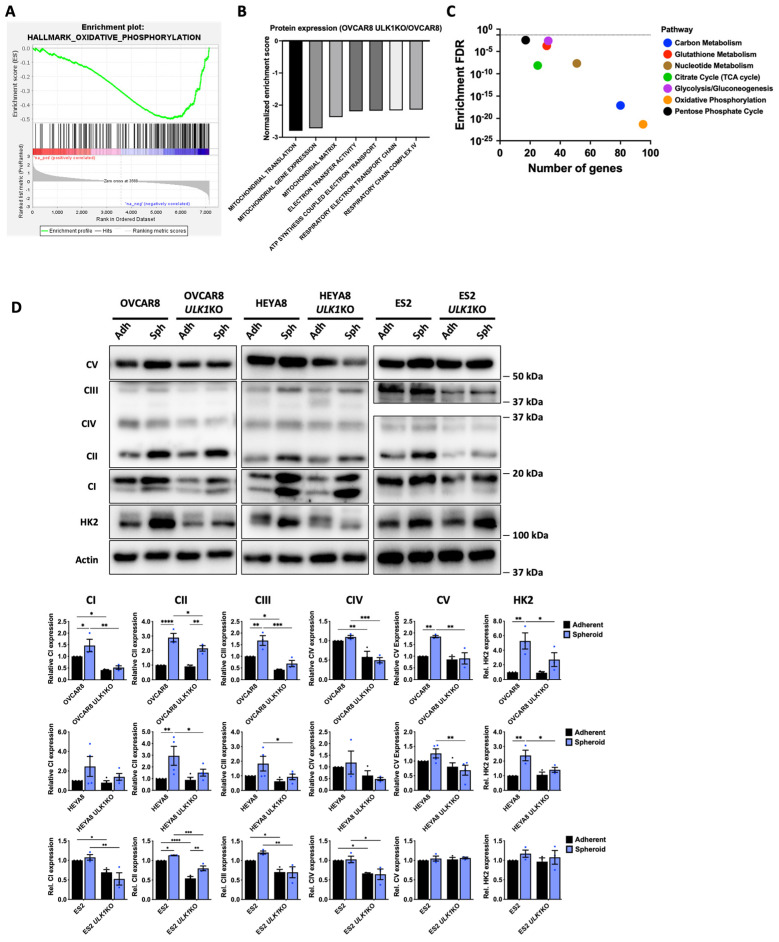
*ULK1* loss remodels OXPHOS protein expression in EOC spheroids. (**A**) GSEA Hallmarks, (**B**) GSEA Biological Processes, and (**C**) KEGG pathway analysis revealed altered metabolism and mitochondrial processes in EOC *ULK1*KO spheroids, including oxidative phosphorylation. Dotted line reflects an FDR of 0.05. (**D**) OVCAR8, HEYA8, and ES2 parental and *ULK1*KO cells were seeded in adherent and spheroid culture. Protein lysates were harvested 72 h after seeding for Western blot analysis for oxidative phosphorylation complex proteins and glycolysis proteins (HK2). Densitometric analysis of oxidative phosphorylation complex (CV, CIII, CIV, CII, CI) and HK2 relative to expression in parental adherent conditions. Data displayed as mean ± SEM; two-way ANOVA followed by Šidák’s multiple comparisons test (*N* = 3–4 biological replicates), * *p* < 0.05, ** *p* < 0.01, *** *p* < 0.001, **** *p* < 0.0001. Original western blots are presented in File S1.

Analysis of previously generated label-free protein mass spectrometry of OVCAR8 and OVCAR8 *ULK1*KO spheroids [8] revealed striking decreases in signatures related to oxidative phosphorylation (OXPHOS) and numerous mitochondrial and metabolic processes in OVCAR8 *ULK1*KO spheroids (Figure 4A–C). To validate these findings, and to test whether *ULK1* loss carries metabolic consequences, we first assessed the protein abundance of five representative OXPHOS complex subunits by immunoblot. Several OXPHOS complexes were significantly reduced under both adherent and spheroid conditions among all three *ULK1*KO lines. Notably, complex V (ATP synthase), essential for ATP production [43], was decreased in OVCAR8 and HEYA8 *ULK1*KO spheroids. Complexes I and IV were reduced in OVCAR8 and ES2 *ULK1*KO spheroids, while reductions in complexes II and III were observed among all three EOC cell line *ULK1*KO spheroid conditions. To further assess mitochondrial protein abundance, we examined outer mitochondrial membrane proteins: TOM20 was significantly reduced in OVCAR8 and ES2 *ULK1*KO spheroids, while VDAC was significantly reduced across all *ULK1*KO spheroid models (Supplementary Figure S3A,B). Because ULK1 perturbation has been reported to shift cells between glycolysis and OXPHOS in a cell-line-specific manner [24,25], we also examined a subset of representative glycolytic proteins (Figure 4D; Supplementary Figure S4A). Among all three EOC *ULK1*KO spheroids, hexokinase-2 (HK2), the rate-limiting enzyme of glycolysis, was the only glycolytic enzyme significantly decreased by both immunoblot (Figure 4D; Supplementary Figure S4A) and proteomics (Supplementary Figure S4B), with no consistent changes observed for other glycolysis components.

**Figure 5:**
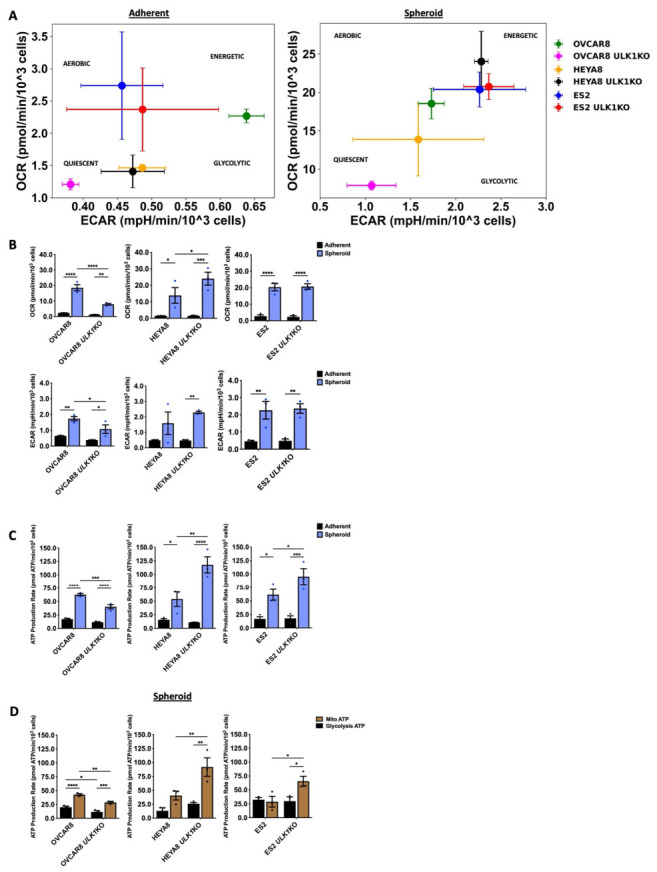
*ULK1* loss rewires bioenergetics in EOC spheroids. (**A**) OCR–ECAR phenograms for adherent (**left**) and spheroid (**right**) cultures. Basal oxygen consumption rate (OCR; pmol/min/10^3^ cells) and extracellular acidification rate (ECAR; mpH/min/10^3^ cells) are plotted as mean ± SEM (error bars) for each line (color-coded legend). Quadrants denote bioenergetic states (Aerobic, Energetic, Glycolytic, Quiescent) as determined by Agilent Seahorse XF Real-Time ATP Rate Assay software (S7888-10012, Agilent Technologies). (**B**) OCR and ECAR values for EOC cells in adherent vs. spheroid culture. Data displayed as mean ± SEM (*N* = 3); two-way ANOVA followed by Šidák’s multiple comparisons test, * *p* < 0.05, ** *p* < 0.01, *** *p* < 0.001, **** *p* < 0.0001. (**C**) Total ATP production rate (pmol ATP/min/10^3^ cells) in adherent and spheroid conditions was derived using the Agilent Seahorse XF Real-Time ATP Rate Assay Kit. Data displayed as mean ± SEM (*N* = 3); two-way ANOVA followed by Šidák’s multiple comparisons test, * *p* < 0.05, ** *p* < 0.01, *** *p* < 0.001, **** *p* < 0.0001. (**D**) Partitioning of ATP production rate (pmol ATP/min/10^3^ cells) into mitochondrial ATP and glycolytic ATP in spheroid conditions. Data displayed as mean ± SEM (*N* = 3); two-way ANOVA followed by Šidák’s multiple comparisons test, * *p* < 0.05, ** *p* < 0.01, *** *p* < 0.001, **** *p* < 0.0001.

Next, we performed Seahorse ATP Rate Assays to assess *ULK1* loss effects on ATP production and net OXPHOS and glycolysis rates. Relative to adherent culture, spheroids showed higher OCR and ECAR overall. OVCAR8 *ULK1*KO spheroids displayed reduced OCR and ECAR, HEYA8 *ULK1*KO spheroids showed increased OCR with unchanged ECAR, but ES2 *ULK1*KO spheroids exhibited no change in both OCR and ECAR (Figure 5A,B). As a net result, ATP production rate was significantly reduced in OVCAR8 *ULK1*KO spheroids, while this was increased in both HEYA8 and ES2 *ULK1*KO spheroids (Figure 5C). These changes aligned with mitochondrial-derived ATP production, which was decreased in OVCAR8 *ULK1*KO yet increased in HEYA8 and ES2 *ULK1*KO spheroids (Figure 5D). Collectively, *ULK1* loss remodels EOC spheroid metabolism, producing broad reductions in OXPHOS complex expression, altered mitochondrial membrane protein marker abundance, and selective loss of HK2. However, the functional consequences of these changes were not uniform, indicating that *ULK1* loss reshapes mitochondrial and metabolic homeostasis differently across ovarian cancer spheroid models.

### 3.5. Therapeutic Potential of Targeting ULK1 and OXPHOS in EOC

**Figure 6:**
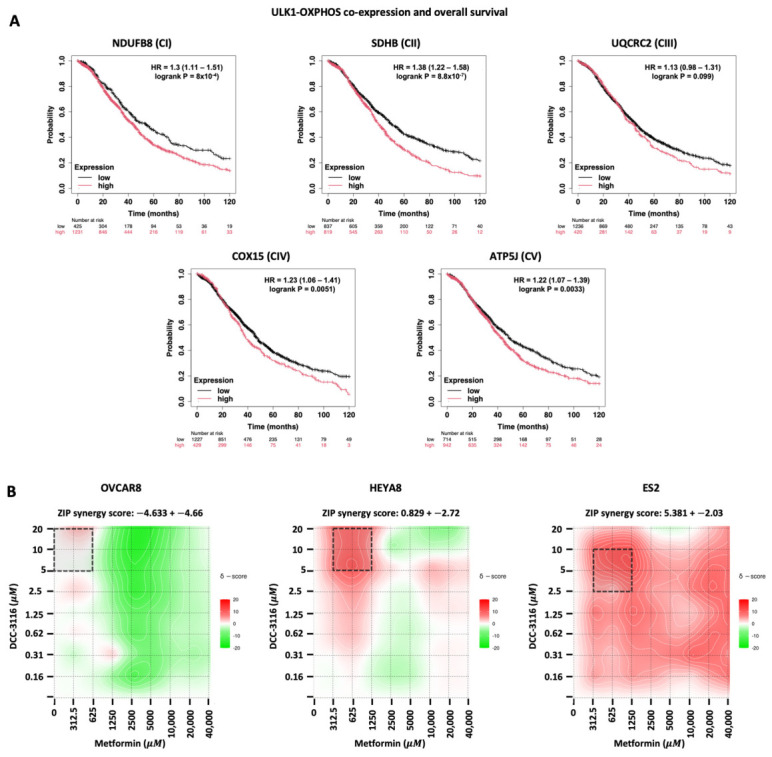
Therapeutic potential of targeting ULK1 and OXPHOS in EOC. (**A**) Correlation of high ULK1 and OXPHOS complex subunit gene expression with overall survival (*N* = 1656) among ovarian cancer patients, regardless of histotype and stage, using TCGA and GEO gene expression microarray datasets from the online tool accessed at www.kmplot.com/ovar (accessed on 10 November 2025). Hazard ratios and log-rank tests indicate a significantly worse prognosis due to high ULK1 and OXPHOS expression in ovarian tumors. (**B**) ZIP synergy scores (ZSS) are presented as mean ± SEM (*N* = 3, with 3 technical replicates per biological replicate). Heat maps display the average synergy scores from biological replicates, where red indicates synergistic interaction (ZSS > 10), white represents additive effects (−10 < ZSS < 10), and green indicates antagonism (ZSS < −10). Regions enclosed by dashed-lined boxes represent areas with the greatest degree of synergy. Cells were seeded 24 h prior to 72-h combination treatment with DCC-3116 and metformin, using a matrix of concentrations ranging from 0 to 20 μM and 0 to 40,000 μM, respectively. Cell viability was assessed using Cell Titer-Glo as an indirect measure of viability.

Given the OXPHOS metabolic dependencies we observed using Seahorse analyses, we assessed ULK1 and OXPHOS complex co-expression in publicly available ovarian cancer patient gene expression datasets. Survival analysis revealed that high co-expression of ULK1 with OXPHOS subunit genes–NDUFB8 (Complex I), SDHB (Complex II), UQCRC2 (Complex III), COX15 (Complex IV), and ATP5J (Complex V)–was associated with significantly decreased overall survival (Figure 6A), supporting a pro-tumorigenic ULK1-OXPHOS axis. To assess potential synergism between combined ULK1 and OXPHOS inhibition, we performed drug combination matrix assays, evaluating 96 unique concentration combinations between DCC-3116 (ULK1 inhibitor) and metformin (complex I inhibitor) in spheroid culture of OVCAR8, HEYA8 and ES2 cell lines. Using Synergy Finder, we calculated the Zero Interaction Potency Synergy Score (ZSS) based on multi-dose and multi-drug combination response data [39,40]. When averaged across all 96 drug combinations, the ZSS for OVCAR8, HEYA8, and ES2 spheroids were within the additive range (i.e., −10 < BSS < 10; Figure 6B). However, distinct regions of synergy were observed within the heat maps, uniformly at medium to high concentrations of DCC-3116 combined with low concentrations of metformin among the three cell lines, with increasing levels of combination potency going from OVCAR8 to HEYA8 to ES2 spheroids (Figure 6B). Within these regions, individual combination synergy scores ≥ 10 were identified for HEYA8 and ES2 spheroids (Supplementary Tables S1–S3). Together, these findings nominate the ULK1–OXPHOS axis as a potential therapeutic vulnerability in advanced EOC.

## 4. Discussion

To the best of our knowledge, we are the first to define non-canonical functions of ULK1 in EOC spheroids, revealing therapeutic vulnerabilities tied to ULK1-mediated mitochondrial homeostasis. These findings extend from prior work on its pro-tumorigenic role in EOC metastasis beyond its established function in autophagy initiation to also govern mitochondrial homeostasis and metabolic adaptation. We show that ULK1 has dichotomous roles in either protecting mitochondria from degradation or promoting their clearance through mechanisms that can be uncoupled from canonical autophagy machinery. Although *ULK1* loss broadly suppresses OXPHOS complex proteins and HK2 expression, ATP production differed across EOC spheroid models. Consistent with this, drug combination matrices suggested potential synergy between the ULK1 inhibitor DCC-3116 and low-dose metformin, underscoring a new, targetable ULK1-OXPHOS vulnerability in metastatic EOC.

Beyond its well-characterized role in initiating bulk autophagy under nutrient deprivation, ULK1 is a key regulator of selective autophagy pathways, including mitophagy. Mitophagy is a canonical autophagic process that mediates the recognition and removal of damaged or dysfunctional mitochondria through LC3 conjugation machinery and autophagosome formation, thereby supporting metabolic fitness and stress tolerance [16]. ULK1 regulates mitophagy through both transcriptional and post-translational mechanisms, acting downstream of AMPK and upstream of receptor-mediated mitochondrial engulfment [22,27]. Thus, we initially speculated that ULK1 would be required for mitophagy in EOC spheroids, also since another study demonstrated that ULK1 overexpression enhances mitophagy in ES2 cells, while *ULK1* knockdown attenuates this process [23]. Indeed, we found that ULK1 was required for mitochondrial degradation in HEYA8 and ES2 spheroids, yet paradoxically, *ULK1* loss in OVCAR8 spheroids enhanced mitochondrial degradation. Most importantly, genetic disruption of core autophagy machinery via *ATG5* and *ATG7* knockdown revealed that regulation of mitochondrial degradation in EOC spheroids proceeds even when canonical autophagy is impaired. These findings support a model in which ULK1 regulates mitochondrial degradation in a manner that can be uncoupled from ATG5/7-dependent canonical autophagy.

Cancer cells can adapt to impaired mitophagy when core autophagy is compromised, and several lysosome-directed mitochondrial quality-control pathways could potentially explain the phenotypes observed. One alternative is the formation of mitochondria-derived vesicles (MDVs), which transport damaged mitochondrial components to lysosomes independently of LC3 and canonical mitophagic mechanisms [44,45]. Another reported route involves Golgi-derived membranes contributing to autophagosomes that engulf mitochondria, as shown in cardiomyocytes [46]. The enrichment of protein secretion and Golgi-associated vesicle signatures in our OVCAR8 *ULK1*KO spheroid proteomics suggests that *ULK1* loss occurs alongside broader changes in vesicular trafficking biology. ARF1 was strongly represented within the protein secretion signature, which is notable given its role in Golgi-associated vesicle formation and endosomal trafficking [47,48,49,50]. In this context, the ability of BFA to partially phenocopy the enhanced mitochondrial reporter degradation observed in OVCAR8 *ULK1*KO spheroids supports the possibility that altered membrane trafficking may contribute to mitochondrial delivery to lysosomes. However, because BFA broadly disrupts ARF-GEF/ARF-dependent Golgi and secretory trafficking [42], these findings should not be interpreted as evidence that ARF1 directly mediates mitochondrial degradation. Rather, they suggest that the enhanced lysosome-associated mitochondrial degradation observed in OVCAR8 *ULK1*KO spheroids occurs in the setting of altered Golgi/secretory trafficking and remains sensitive to perturbation of this pathway.

More broadly, the persistence or enhancement of mitochondrial reporter degradation under *ATG5/7*-depleted conditions suggests that mitochondrial delivery to acidic compartments in EOC spheroids is not strictly dependent on canonical autophagy machinery. This was particularly evident in OVCAR8 *ULK1*KO spheroids, where enhanced mitochondrial degradation persisted despite *ATG5/7* knockdown and was attenuated by CQ. In HEYA8 and ES2 spheroids, *ATG5/7* knockdown also increased mitochondrial reporter degradation, indicating that disruption of canonical autophagy can unmask or enhance alternative mitochondrial degradation responses. Together, these findings suggest that ULK1 can either promote or restrain lysosome-associated mitochondrial degradation in EOC spheroids through mechanisms that can be uncoupled from ATG5/7-dependent canonical autophagy. However, the divergent effects of *ULK1* loss and *ATG5/7* knockdown across OVCAR8, HEYA8, and ES2 spheroids make it unlikely that a single mechanism explains mitochondrial degradation across all EOC models. Although candidate lysosome-directed trafficking routes, including MDV-, Golgi-, or endosome-associated pathways, may contribute to these phenotypes, the precise mechanisms remain undefined.

While this work supports ULK1 as a regulator of mitochondrial homeostasis and metabolic adaptation in EOC spheroids, a major theme emerging from these studies is that the consequences of *ULK1* loss differ across EOC spheroid models, particularly when differentiating OVCAR8 from HEYA8/ES2 spheroids. Baseline mitochondrial degradation also did not clearly predict the response to *ULK1* loss, as parental OVCAR8 and ES2 spheroids displayed similar mitochondrial degradation levels despite opposite *ULK1*KO-associated phenotypes, while HEYA8 spheroids showed higher baseline mitochondrial degradation. Similarly, mitochondrial protein abundance did not directly parallel mitochondrial reporter degradation status. Although HEYA8 and ES2 *ULK1*KO spheroids showed reduced mitochondrial reporter degradation, VDAC and several OXPHOS complexes were still reduced in *ULK1*KO spheroids, with TOM20 also reduced in ES2 *ULK1*KO spheroids. These findings suggest that mitochondrial reporter degradation, mitochondrial protein abundance, and bioenergetic output do not map linearly across EOC spheroid models. Together, these observations suggest that no single mitochondrial readout fully explains why *ULK1* loss produces divergent mitochondrial degradation phenotypes. Instead, differences in proliferative capacity, mitochondrial stress state, and spheroid growth behavior may influence whether ULK1 primarily acts to preserve mitochondrial integrity or support bioenergetic adaptation. Indeed, we have shown previously that OVCAR8 parental spheroids do not expand in viable cell number beyond three days in suspension, while viable cell number significantly increases over time in HEYA8 and ES2 spheroids. In fact, *ULK1* loss significantly reduces spheroid cell number in all three of these lines, with HEYA8 and ES2 *ULK1*KO spheroid cells slowly increasing over time as compared with parental lines, whereas OVCAR8 *ULK1*KO fail to retain viability [8]. These characteristics imply underlying dormant (OVCAR8) versus proliferative (HEYA8 and ES2) phenotypes separating these two spheroid models. This distinction was further supported by our organoid studies, where growth capacity increased from OVCAR8 to HEYA8 to ES2, and *ULK1* loss impaired organoid growth in OVCAR8 and HEYA8 but not ES2 [8]. Similarly, in vivo mouse xenograft studies showed that HEYA8 cells produced more aggressive disease than OVCAR8 cells for parental cell lines, as reflected by a significantly shorter experimental endpoint, whereas *ULK1* loss had a more pronounced effect in attenuating disease progression in OVCAR8 [8]. Additionally, we have shown that parental OVCAR8 and HEYA8 spheroids possess significantly different levels of autophagy and mitophagy and altered mitochondrial networks: OVCAR8 spheroids have higher autophagy, lower mitophagy, and a higher proportion of fragmented mitochondria relative to HEYA8 spheroids [29]. Given these properties, we speculate that OVCAR8 spheroids adopt a dormancy-like state that prioritizes cell viability over expansion and, upon *ULK1* loss, shift toward enhanced mitochondrial degradation, reduced mitochondrial ROS, and reduced ATP production. In contrast, HEYA8 and ES2 spheroids appear to retain a more proliferative and metabolically active state in which *ULK1* loss reduces mitochondrial degradation while maintaining or increasing mitochondrial ATP production, potentially at the cost of elevated mitochondrial ROS. Thus, the divergent effects of *ULK1* loss on mitochondrial degradation, ROS status, and bioenergetic output may reflect underlying differences in spheroid growth state and mitochondrial stress adaptation, rather than a single shared ULK1-dependent mechanism across EOC models.

Our ROS findings should be interpreted as correlative rather than causal. Increased mitochondrial degradation in OVCAR8 *ULK1*KO spheroids corresponded with reduced mitochondrial ROS, whereas reduced mitochondrial degradation in HEYA8 and ES2 *ULK1*KO spheroids corresponded with elevated mitochondrial ROS. This inverse relationship is consistent with the role of mitochondrial quality-control pathways in limiting the persistence of damaged or dysfunctional mitochondria. However, mitochondrial ROS are also strongly influenced by electron transport chain activity, as electrons moving through the ETC can prematurely reduce molecular oxygen to generate superoxide, including at Complexes I and III [51]. Therefore, the ROS phenotypes observed here likely reflect the combined influence of altered mitochondrial degradation and cell line-specific respiratory metabolism. Future studies using ROS scavengers, mitochondrial antioxidants, or targeted modulation of respiratory chain activity will be required to define whether mitochondrial ROS is a cause or consequence of ULK1-dependent mitochondrial turnover, altered metabolism, or both.

Despite the absence of a unified mechanism in mitochondrial degradation, ROS production, and ATP partitioning across EOC models, ablation of *ULK1* revealed a shared therapeutic vulnerability. We previously observed reduced ULK1 activity, as measured by decreased p-BECN1 (S30), and reduced EOC spheroid cell viability following treatment with 1 µM DCC-3116 [8]. Here, we show that dual inhibition of ULK1 and mitochondrial Complex I produced reproducible “synergy hotspots” at low concentrations of metformin combined with medium-to-high concentrations of DCC-3116 across EOC spheroids. These findings suggest that, despite cell line-specific metabolic remodeling following *ULK1* loss, pharmacological ULK1 inhibition can sensitize EOC spheroids to Complex I inhibition within defined dose windows. Metformin, a widely prescribed biguanide for type II diabetes that inhibits mitochondrial Complex I, has been evaluated in multiple cancer clinical trials, including for ovarian cancer [52,53,54,55]. DCC-3116 is a selective ULK1 inhibitor currently undergoing clinical evaluation (NCT04892017; NCT05957367). Using these two pharmacologic agents together may offer a clinically meaningful combination. Notably, anticancer applications of metformin use are oftentimes limited due to high dose-related toxicities; our observation that lower metformin concentrations are sufficient to achieve reduced EOC spheroid cell viability when paired with DCC-3116 is encouraging [55,56]. Because both agents are orally bioavailable, DCC-3116 has existing clinical PK/PD development, and metformin has a well-established safety profile, this combination is certainly feasible to explore further. However, additional in vitro validation and dose optimization will be required before advancing to in vivo studies.

While bona fide regions of synergy (ZSS ≥ 10) were detected for drug-treated HEYA8 and ES2 spheroids, this same level of synergy was not achieved for OVCAR8 spheroids. Again, this divergence may reflect their underlying differences in proliferative capacity and metabolic state. Perhaps this could guide biomarker-driven stratification strategies where highly proliferative EOC tumors would derive greater benefit from combined ULK1 and OXPHOS inhibition. Because we performed our drug synergy studies using ATP-based readouts that reflect metabolic impairment rather than direct cell death, validation with orthogonal viability assays will be required to determine the durability of this drug combination.

## 5. Conclusions

In summary, we define non-canonical roles for ULK1 in ovarian cancer spheroids, showing that it can either promote or restrain lysosome-associated mitochondrial degradation independently of core autophagy machinery. *ULK1* loss remodels both mitochondrial ATP production and ROS status differently across EOC spheroid models. Finally, DCC-3116 treatment with low-dose metformin suggests that dual ULK1–OXPHOS targeting represents a potential therapeutic vulnerability in advanced EOC that warrants further exploration.

## Data Availability

The original contributions presented in this study are included in the article/Supplementary Materials. Further inquiries can be directed to the corresponding author. The raw data supporting the conclusions of this article will be made available by the authors on request.

## Supplementary Materials

The following supporting information can be downloaded at: https://www.mdpi.com/article/10.3390/cancers18111746/s1, Figure S1: Chloroquine treatment reveals lysosome-dependent regulation of mitoR processing in OVCAR8 parental and *ULK1*KO spheroids.; Figure S2: Brefeldin A enhances lysosome-associated mitochondrial reporter processing in OVCAR8 spheroids.; Figure S3. *ULK1* loss reduces mitochondrial protein abundance in EOC spheroids.; Figure S4. Effects of *ULK1* loss on glycolysis protein abundance in EOC cells.; Table S1: ZIP synergy scores matrix for HEYA8 spheroids; Table S2: ZIP synergy scores matrix for ES2 spheroids; Table S3: ZIP synergy scores matrix for OVCAR8 spheroids. File S1. Original western blots.

## Abbreviations

The following abbreviations are used in this manuscript:

ADH	Adherent
AMPK	AMP-activated protein kinase
ARF1	ADP-ribosylation factor 1
ATG5	Autophagy related 5
ATG7	Autophagy related 7
BECN1	Beclin 1
BFA	Brefeldin A
CMV	Cytomegalovirus
CQ	Chloroquine
DMEM/F12	Dulbecco’s Modified Eagle Medium/Nutrient Mixture F-12
ECAR	Extracellular acidification rate
EGFP	Enhanced green fluorescent protein
EOC	Epithelial ovarian cancer
FACS	Fluorescence-activated cell sorting
GEF	Guanine nucleotide exchange factor
GEO	Gene Expression Omnibus
GSEA	Gene set enrichment analysis
HK2	Hexokinase 2
HRP	Horseradish peroxidase
KEGG	Kyoto Encyclopedia of Genes and Genomes
LC3	Microtubule-associated protein 1 light chain 3
MDVs	Mitochondria-derived vesicles
mTORC1	Mechanistic target of rapamycin complex 1
OCR	Oxygen consumption rate
OXPHOS	Oxidative phosphorylation
PBS	Phosphate-buffered saline
PINK1	PTEN-induced kinase 1
RFU	Relative fluorescence units
RIPA	Radioimmunoprecipitation assay
ROS	Reactive oxygen species
RPMI-1640	Roswell Park Memorial Institute 1640 medium
SPH	Spheroid
siULK1	Small interfering RNA targeting ULK1
SPoRTS	Spatial Profiling of Ratiometric Trends in Spheroids
TCGA	The Cancer Genome Atlas
ULA	Ultra-low attachment
ULK1	Unc-51-like kinase 1
*ULK1*KO	*ULK1* knockout
VDAC	Voltage-dependent anion channel
XF	Extracellular flux
ZIP	Zero Interaction Potency
ZSS	ZIP synergy score
