# Supplementary material for: Divergent Role of ULK1 to Balance Mitochondrial Homeostasis and Bioenergetics in Ovarian Cancer Spheroids
Source: Cancers (Basel). 2026 May 27;18(11):1746. doi: 10.3390/cancers18111746 (PMC13255712; doi:10.3390/cancers18111746)

Figure 1

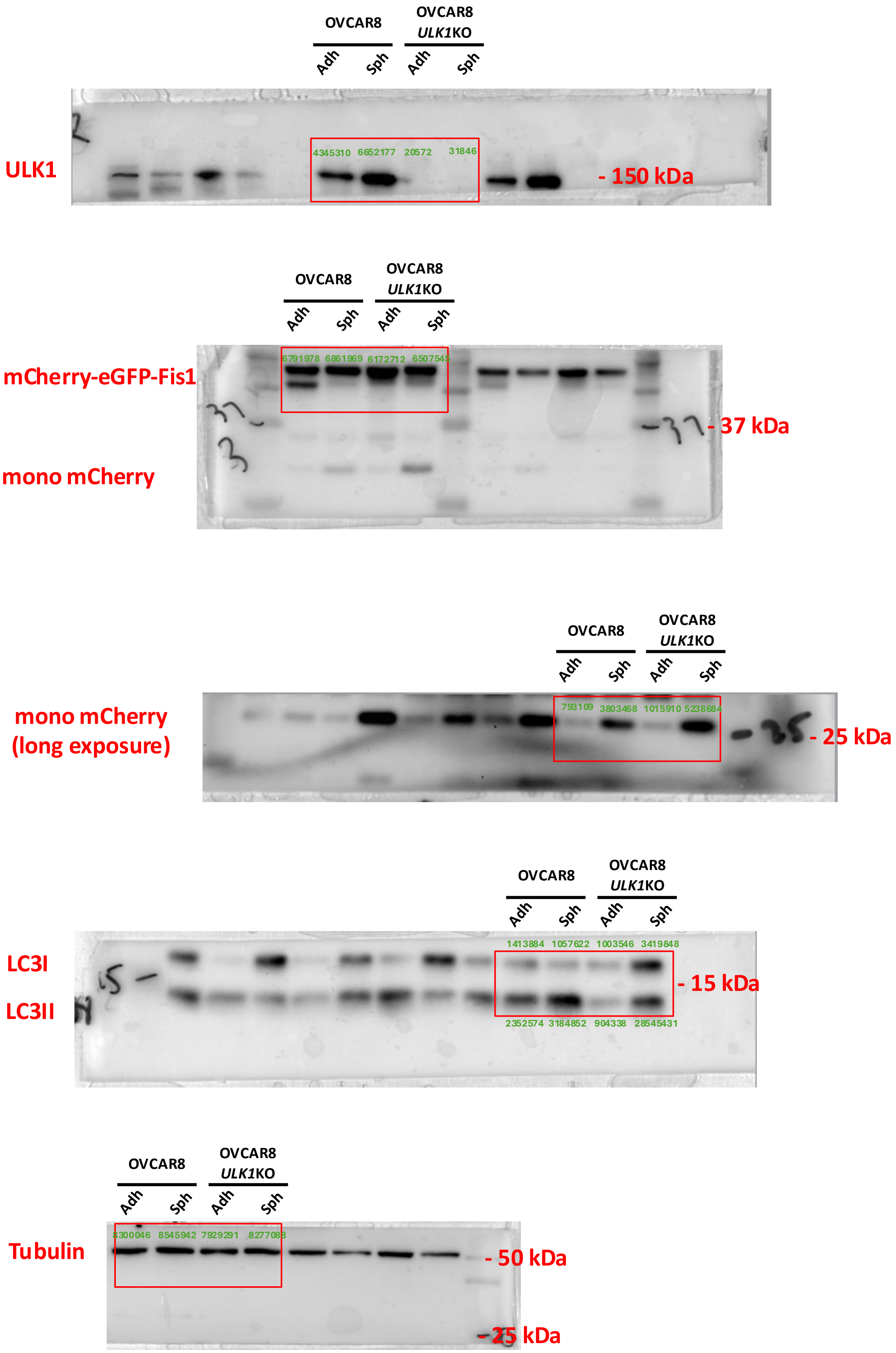

OVCAR8
OVCAR8 ULK1KO
Adh
Sph
Adh
Sph
ULK1
4345310
6652177
20572
31846
- 150 kDa
mCherry-eGFP-Fis1
6791978
6861969
6172712
6507548
- 37 kDa
mono mCherry
mono mCherry (long exposure)
793109
3803468
1015910
5238684
- 25 kDa
LC3I
LC3II
1413884
1057622
1003546
3419848
2352574
3184852
904338
28545431
- 15 kDa
Tubulin
8300046
8545942
7929291
8277088
- 50 kDa
- 25 kDa

Figure 1

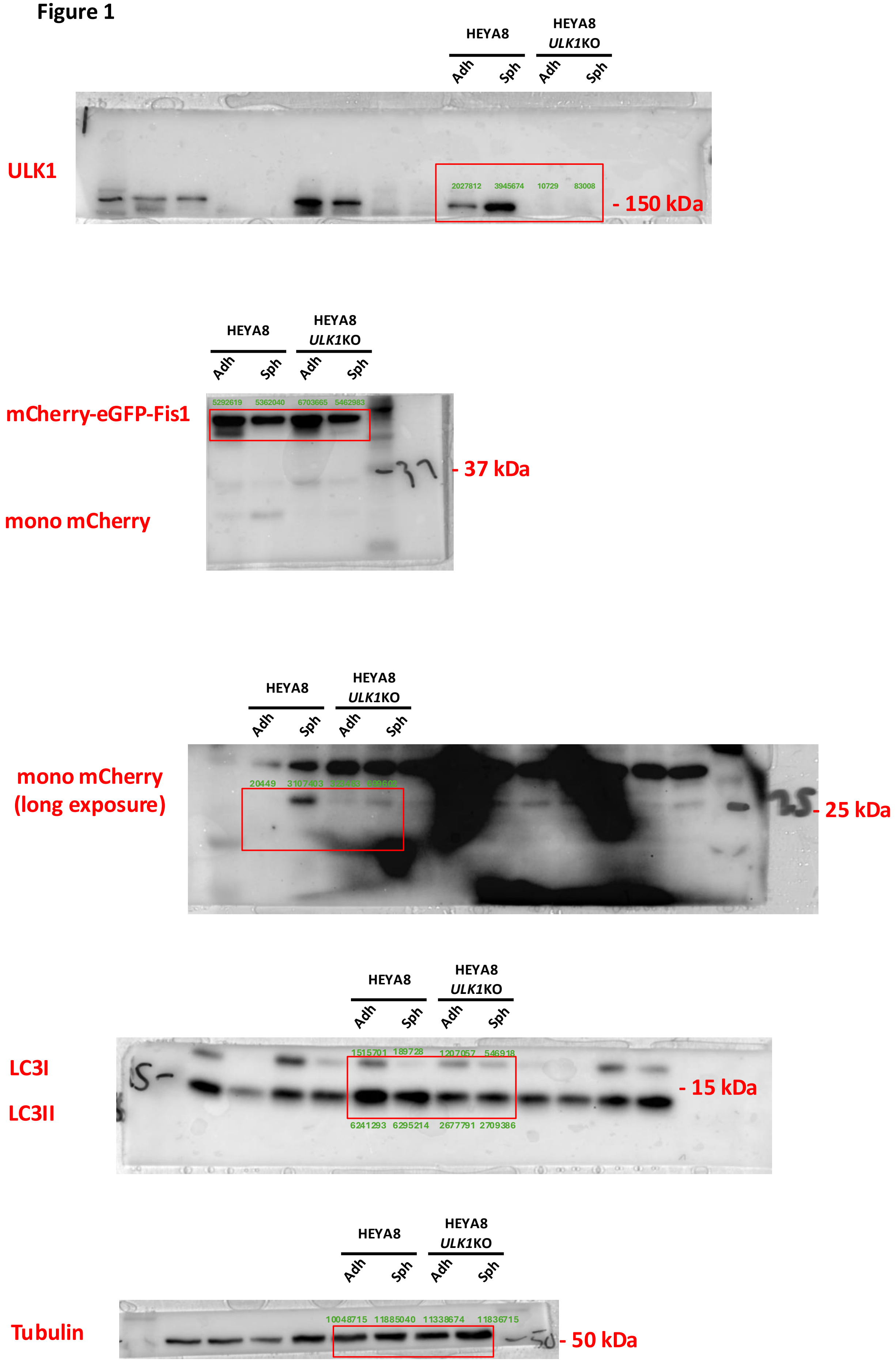
HEYA8
HEYA8 ULK1KO
Adh
Sph
Adh
Sph
ULK1
2027812
3945674
10729
83008
- 150 kDa
mCherry-eGFP-Fis1
5292619
5362040
6703665
5462983
- 37 kDa
mono mCherry
mono mCherry (long exposure)
20449
3107403
- 25 kDa
LC3I
LC3II
1515701
189728
1207057
546918
- 15 kDa
6241293
6295214
2677791
2709386
Tubulin
10048715
11885040
11338674
11836715
- 50 kDa

Figure 1

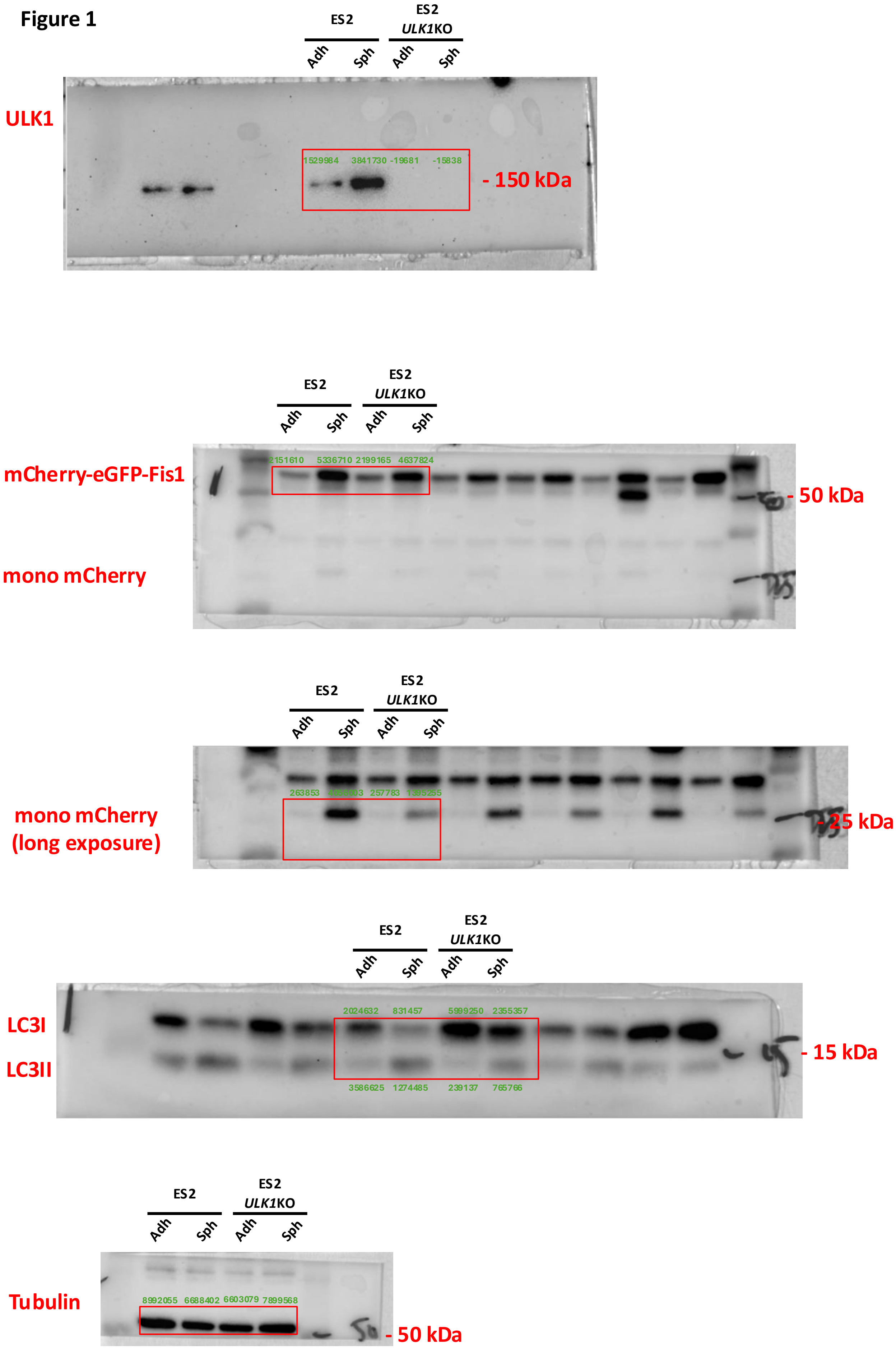

ES2
ES2 ULK1KO
Adh
Sph
Adh
Sph
ULK1
1529984
3841730
-19681
-15838
- 150 kDa
mCherry-eGFP-Fis1
2151610
5336710
2199165
4637824
- 50 kDa
mono mCherry
mono mCherry (long exposure)
263853
257783
1395255
- 25 kDa
LC3I
LC3II
2024632
831457
5999250
2355357
3586625
1274485
239137
765766
- 15 kDa
Tubulin
8992055
6688402
6603079
7899568
- 50 kDa

Figure 2A

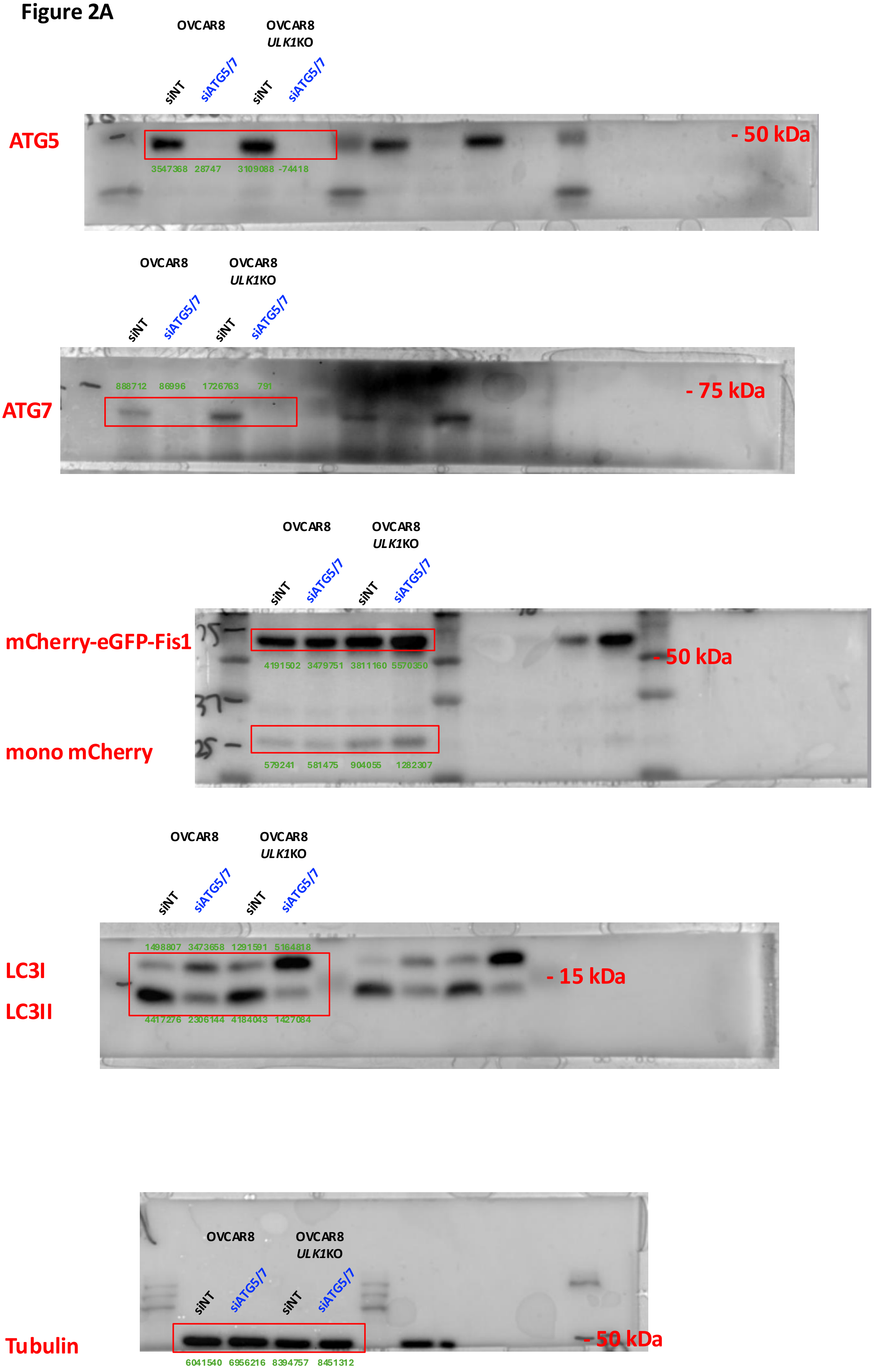

OVCAR8
OVCAR8 ULK1KO
siNT
siATG5/7
siNT
siATG5/7
ATG5
- 50 kDa
3547368 28747 3109088 -74418
OVCAR8
OVCAR8 ULK1KO
siNT
siATG5/7
siNT
siATG5/7
ATG7
- 75 kDa
888712 86996 1726763 791
OVCAR8
OVCAR8 ULK1KO
siNT
siATG5/7
siNT
siATG5/7
mCherry-eGFP-Fis1
- 50 kDa
4191502 3479751 3811160 5570350
mono mCherry
579241 581475 904055 1282307
OVCAR8
OVCAR8 ULK1KO
siNT
siATG5/7
siNT
siATG5/7
LC3I
LC3II
- 15 kDa
1498807 3473658 1291591 5164818
4417276 2306144 4184043 1427084
OVCAR8
OVCAR8 ULK1KO
siNT
siATG5/7
siNT
siATG5/7
Tubulin
- 50 kDa
6041540 6956216 8394757 8451312

Figure 2A

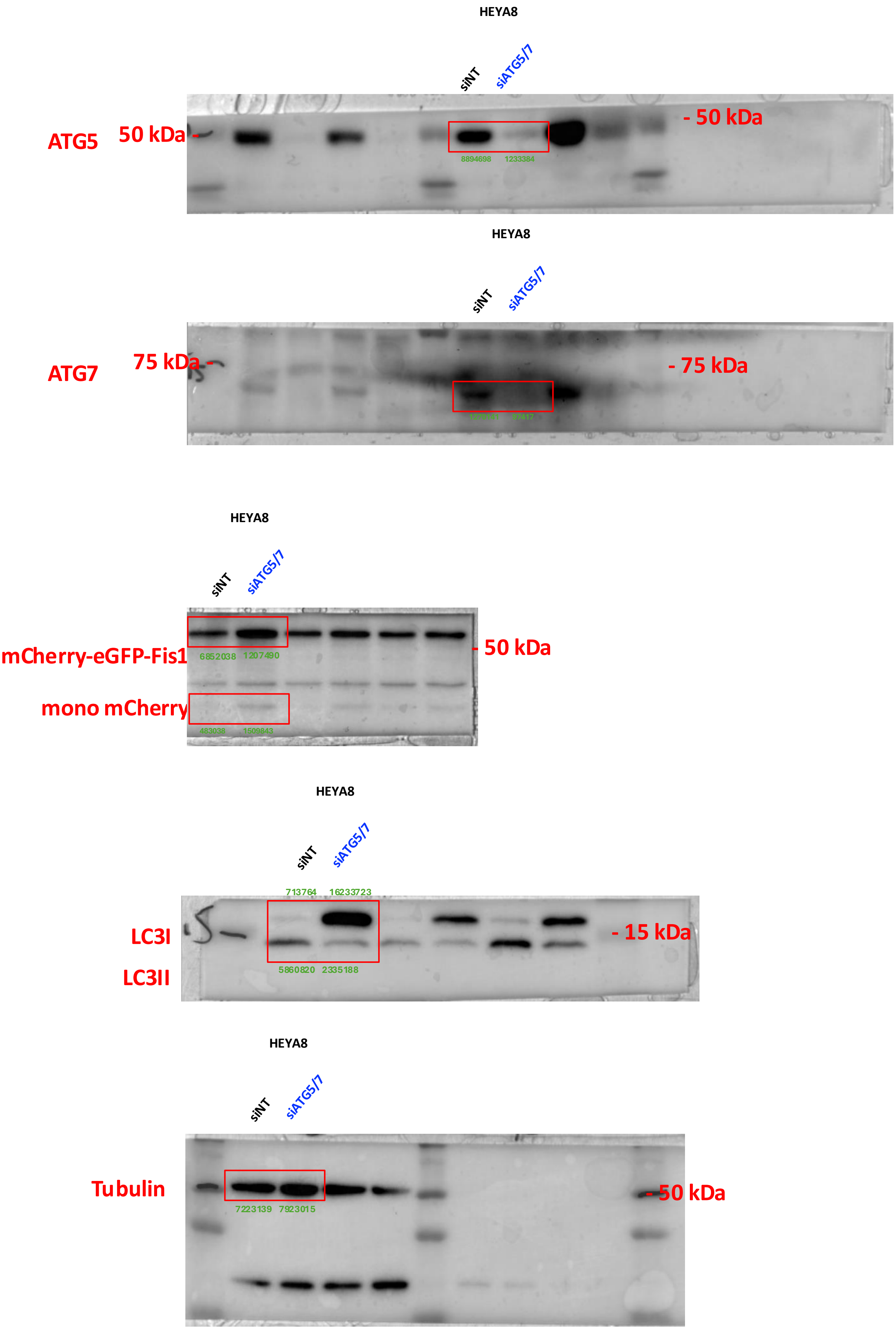

HEYA8
siNT
siATG5/7
ATG5
50 kDa
- 50 kDa
8894698
1233384
HEYA8
siNT
siATG5/7
ATG7
75 kDa
- 75 kDa
1270141
97417
HEYA8
siNT
siATG5/7
mCherry-eGFP-Fis1
mono mCherry
6852038
1207490
483038
1509843
- 50 kDa
HEYA8
siNT
siATG5/7
713764
16233723
LC3I
LC3II
5860820
2335188
- 15 kDa
HEYA8
siNT
siATG5/7
Tubulin
7223139
7923015
-50 kDa

Figure 2A

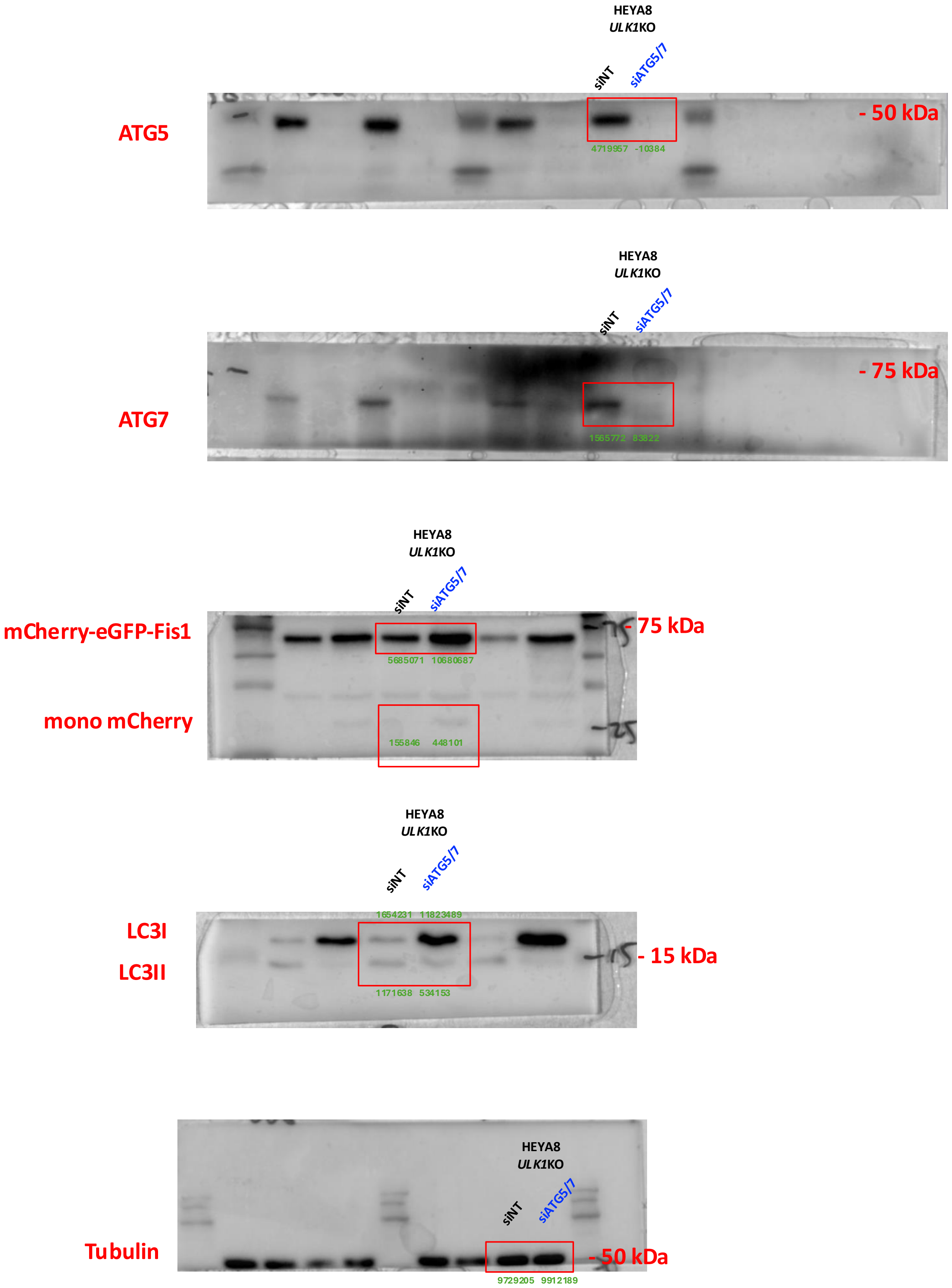

HEYA8
ULK1KO
siNT
siATG5/7
ATG5
- 50 kDa
4719957
-10384
HEYA8
ULK1KO
siNT
siATG5/7
ATG7
- 75 kDa
1565772
83822
HEYA8
ULK1KO
siNT
siATG5/7
mCherry-eGFP-Fis1
- 75 kDa
5685071
10680687
mono mCherry
155846
448101
HEYA8
ULK1KO
siNT
siATG5/7
LC3I
LC3II
1654231
11823489
- 15 kDa
1171638
534153
HEYA8
ULK1KO
siNT
siATG5/7
Tubulin
- 50 kDa
9729205
9912189

Figure 2C

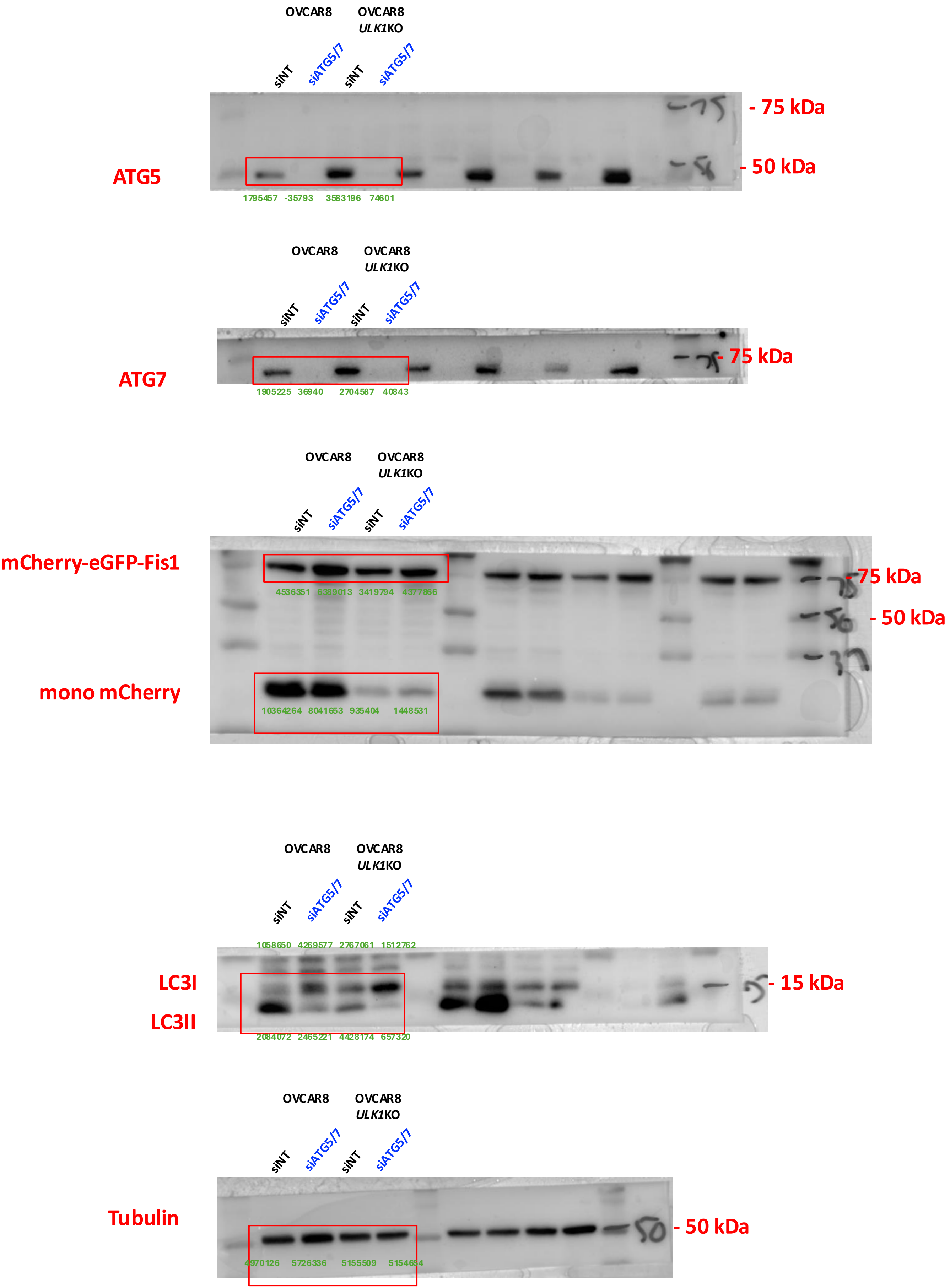

OVCAR8
OVCAR8 ULK1KO
siNT
siATG5/7
siNT
siATG5/7
ATG5
- 75 kDa
- 50 kDa
1795457 -35793 3583196 74601
OVCAR8
OVCAR8 ULK1KO
siNT
siATG5/7
siNT
siATG5/7
ATG7
- 75 kDa
1905225 36940 2704587 40843
OVCAR8
OVCAR8 ULK1KO
siNT
siATG5/7
siNT
siATG5/7
mCherry-eGFP-Fis1
-75 kDa
- 50 kDa
4536351 6389013 3419794 4377866
mono mCherry
10364264 8041653 935404 1448531
OVCAR8
OVCAR8 ULK1KO
siNT
siATG5/7
siNT
siATG5/7
1058650 4269577 2767061 1512762
LC3I
LC3II
- 15 kDa
2084072 2465221 4428174 657320
OVCAR8
OVCAR8 ULK1KO
siNT
siATG5/7
siNT
siATG5/7
Tubulin
- 50 kDa
4970126 5726336 5155509 5154684

Figure 2C

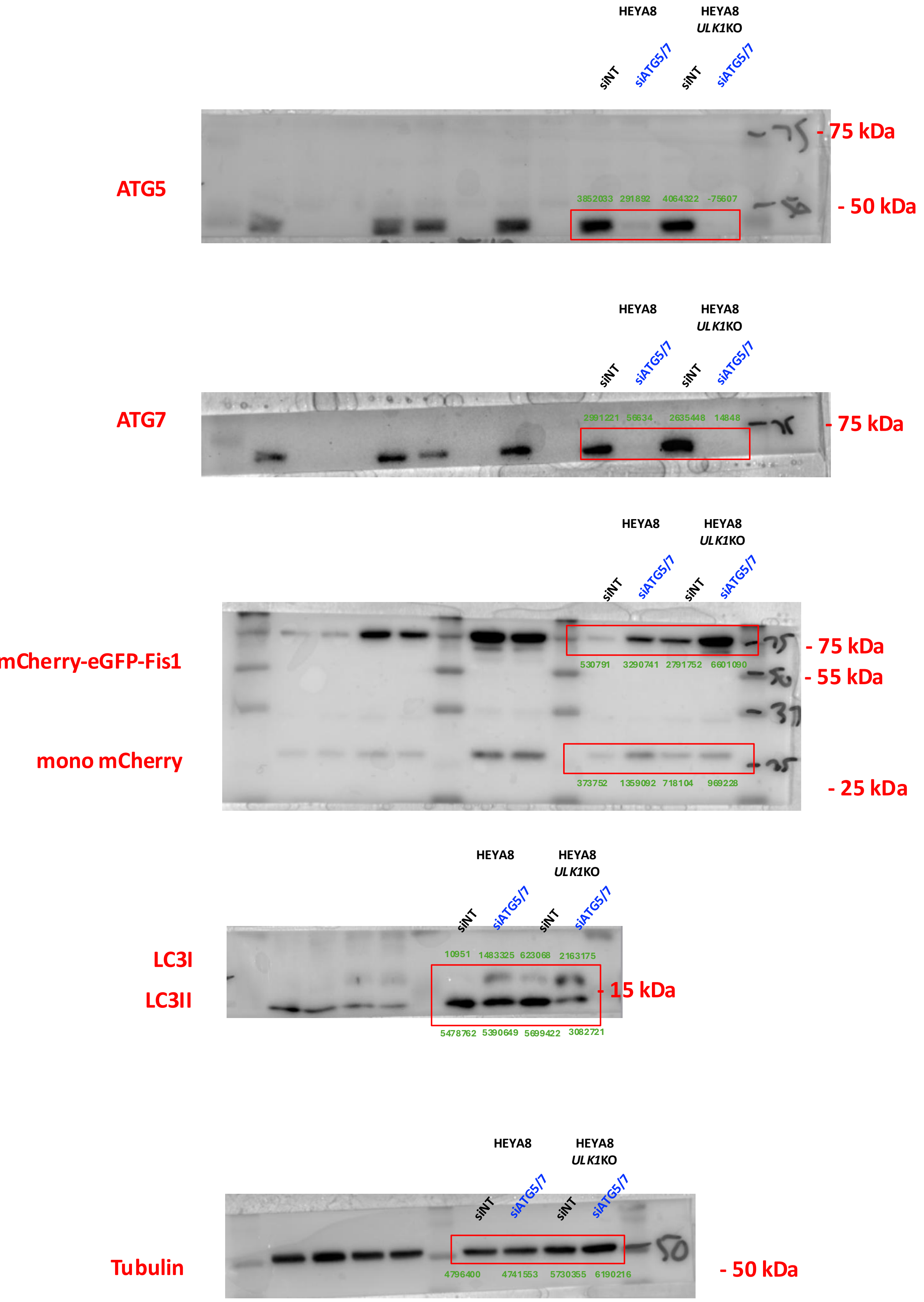
HEYA8
HEYA8 ULK1KO
siNT
siATG5/7
siNT
siATG5/7
ATG5
- 75 kDa
- 50 kDa
3852033 291892 4064322 -75607
HEYA8
HEYA8 ULK1KO
siNT
siATG5/7
siNT
siATG5/7
ATG7
- 75 kDa
2991221 56634 2635448 14848
HEYA8
HEYA8 ULK1KO
siNT
siATG5/7
siNT
siATG5/7
mCherry-eGFP-Fis1
mono mCherry
- 75 kDa
- 55 kDa
- 25 kDa
530791 3290741 2791752 6601090
373752 1359092 718104 969228
HEYA8
HEYA8 ULK1KO
siNT
siATG5/7
siNT
siATG5/7
LC3I
LC3II
- 15 kDa
10951 1483325 623068 2163175
5478762 5390649 5699422 3082721
HEYA8
HEYA8 ULK1KO
siNT
siATG5/7
siNT
siATG5/7
Tubulin
- 50 kDa
4796400 4741553 5730355 6190216

Figure 2E

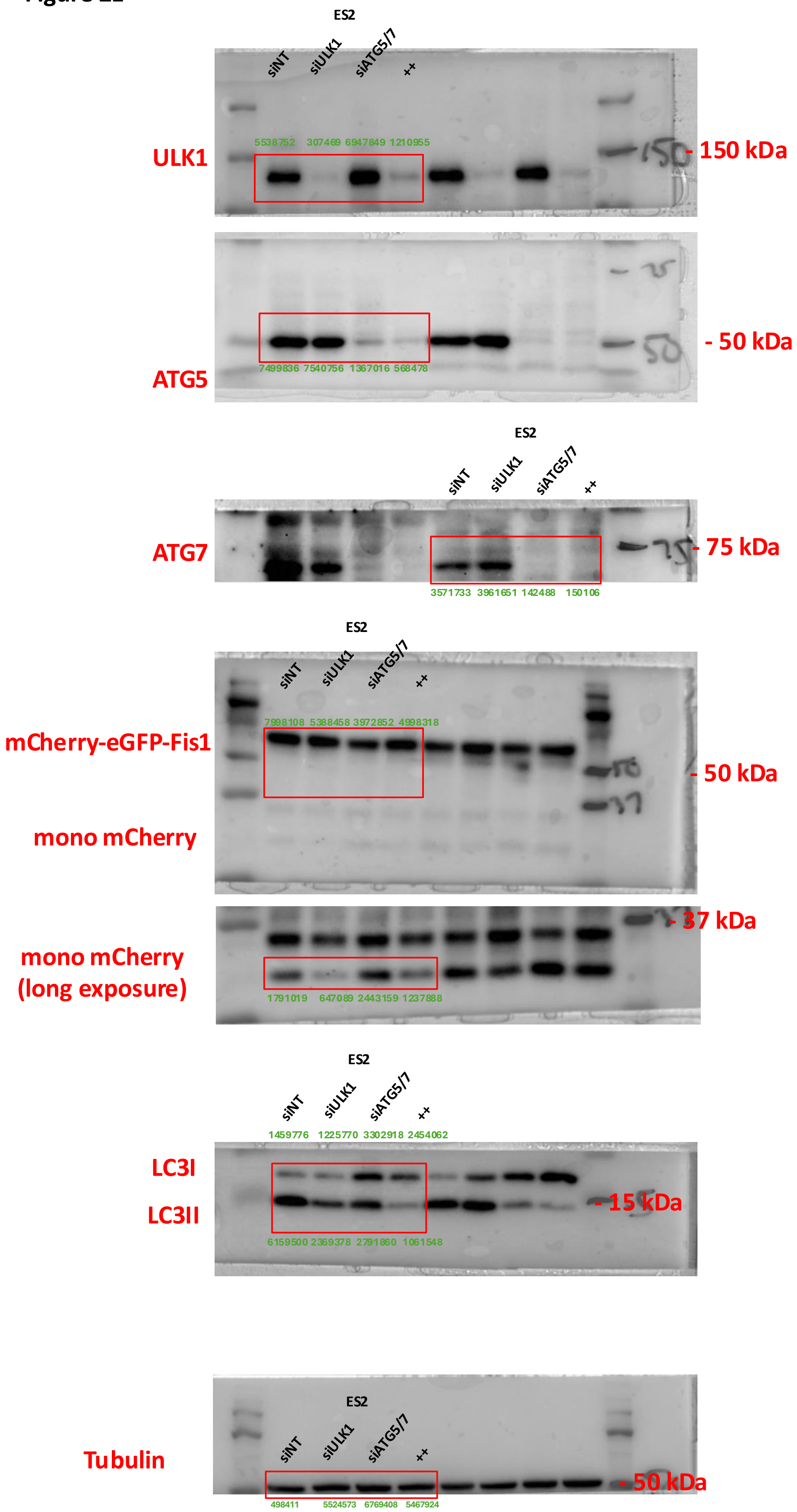

ES2
siNT
siULK1
siATG5/7
++
ULK1
5538752 307469 6947849 1210955
150
- 150 kDa
ATG5
7499836 7540756 1367016 568478
75
50
- 50 kDa
ATG7
3571733 3961651 142488 150106
75
- 75 kDa
mCherry-eGFP-Fis1
7998108 5388458 3972852 4998318
50
37
- 50 kDa
mono mCherry
mono mCherry (long exposure)
1791019 647089 2443159 1237888
- 37 kDa
LC3I
1459776 1225770 3302918 2454062
LC3II
6159500 2369378 2791860 1061548
- 15 kDa
Tubulin
498411 5524573 6769408 5467924
- 50 kDa

Figure 2F

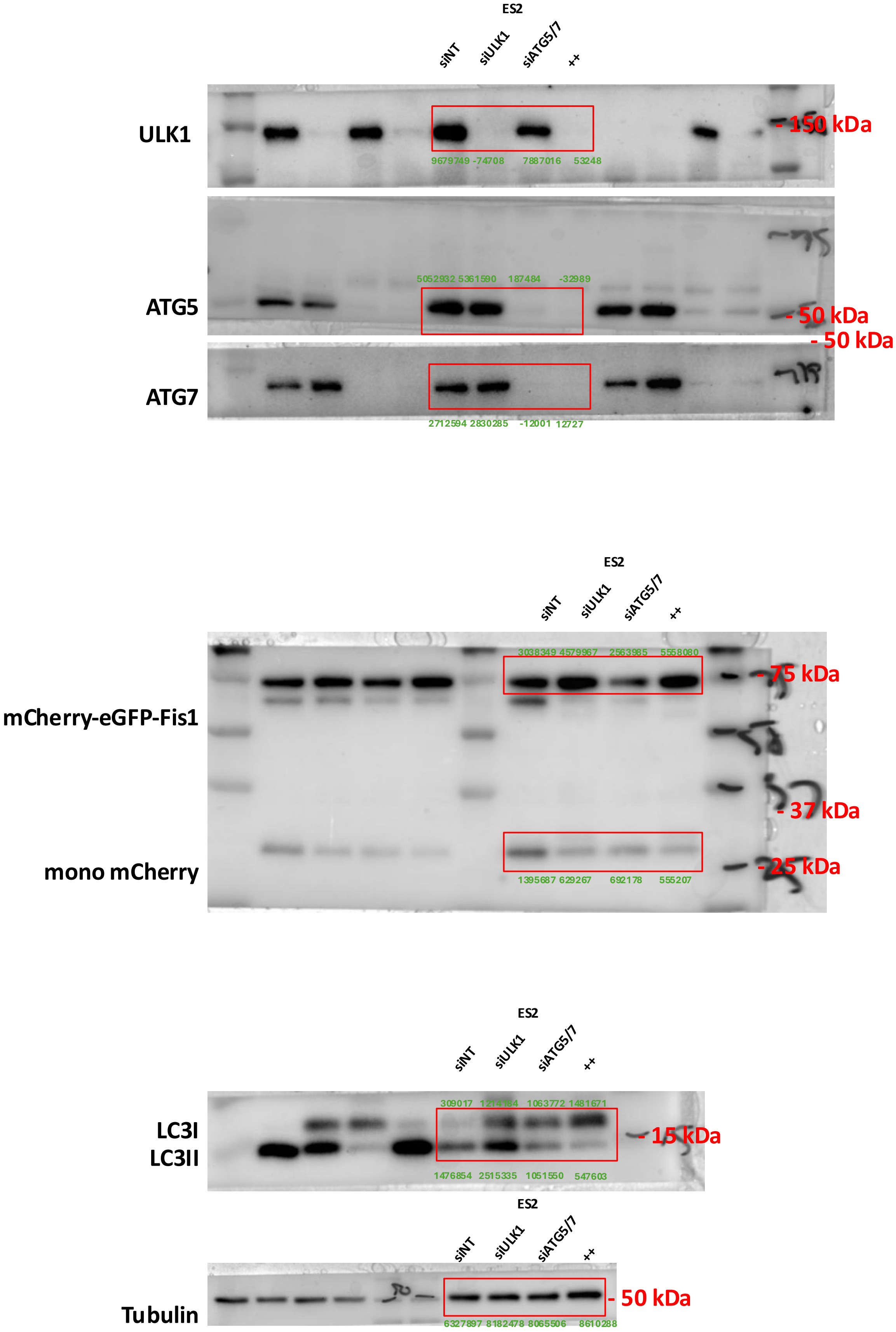

ES2
siNT
siULK1
siATG5/7
++
ULK1
- 150 kDa
9679749 -74708
7887016
53248
ATG5
5052932 5361590
187484
-32989
- 50 kDa
- 50 kDa
ATG7
2712594 2830285
-12001
12727
ES2
siNT
siULK1
siATG5/7
++
mCherry-eGFP-Fis1
3038349 4579967
2563985
5558080
- 75 kDa
- 37 kDa
- 25 kDa
mono mCherry
1395687 629267
692178
555207
ES2
siNT
siULK1
siATG5/7
++
LC3I
LC3II
309017
1214184
1063772 1481671
- 15 kDa
1476854
2515335
1051550
547603
ES2
siNT
siULK1
siATG5/7
++
Tubulin
- 50 kDa
6327897 8182478 8065506
8610288

Figure 4

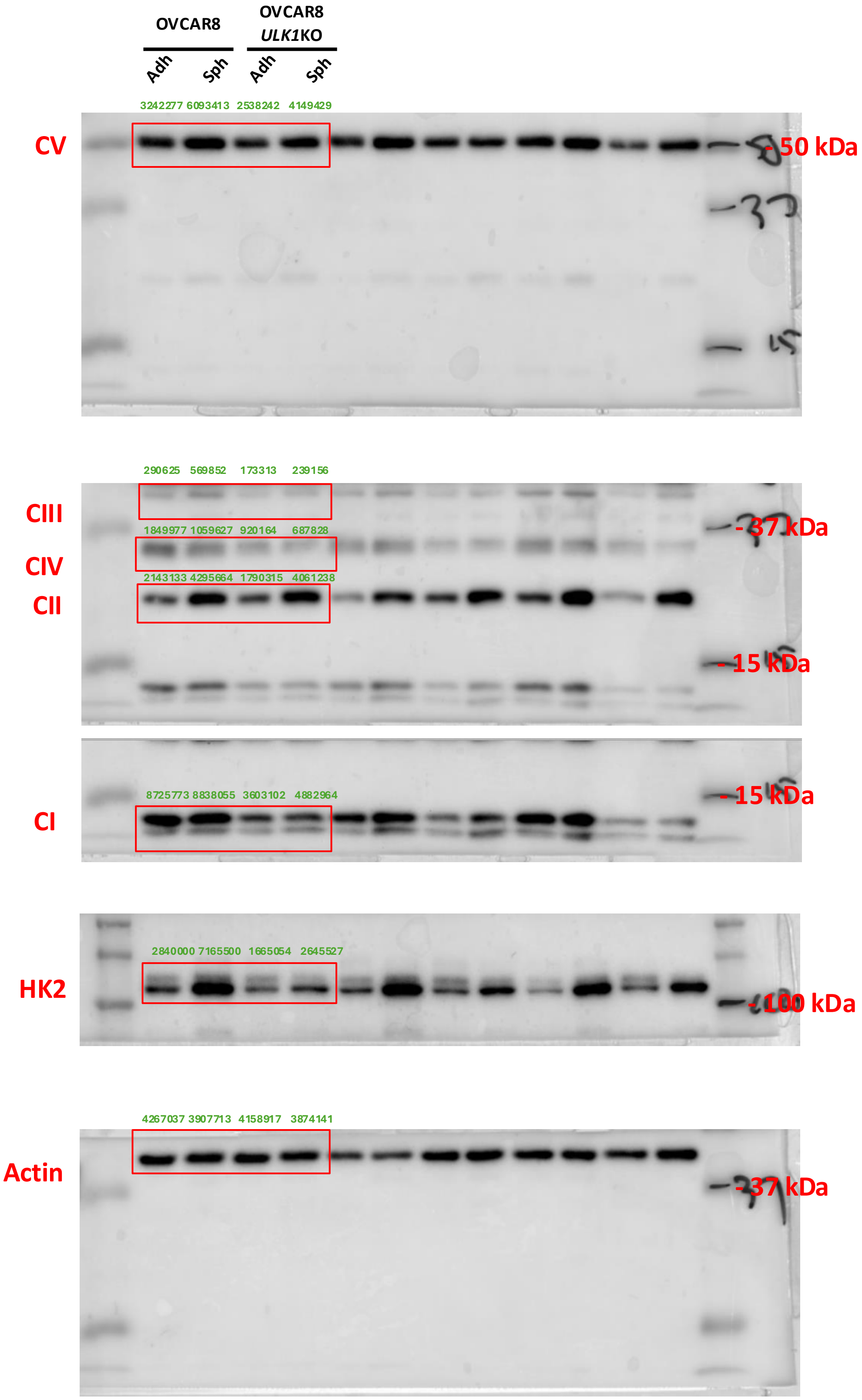
OVCAR8
OVCAR8 ULK1KO
Adh
Sph
Adh
Sph
3242277 6093413 2538242 4149429
CV
50 kDa
37
15
290625 569852 173313 239156
CIII
1849977 1059627 920164 687828
CIV
37 kDa
2143133 4295664 1790315 4061238
CII
15 kDa
8725773 8838055 3603102 4882964
CI
15 kDa
2840000 7165500 1665054 2645527
HK2
100 kDa
4267037 3907713 4158917 3874141
Actin
37 kDa

Figure 4

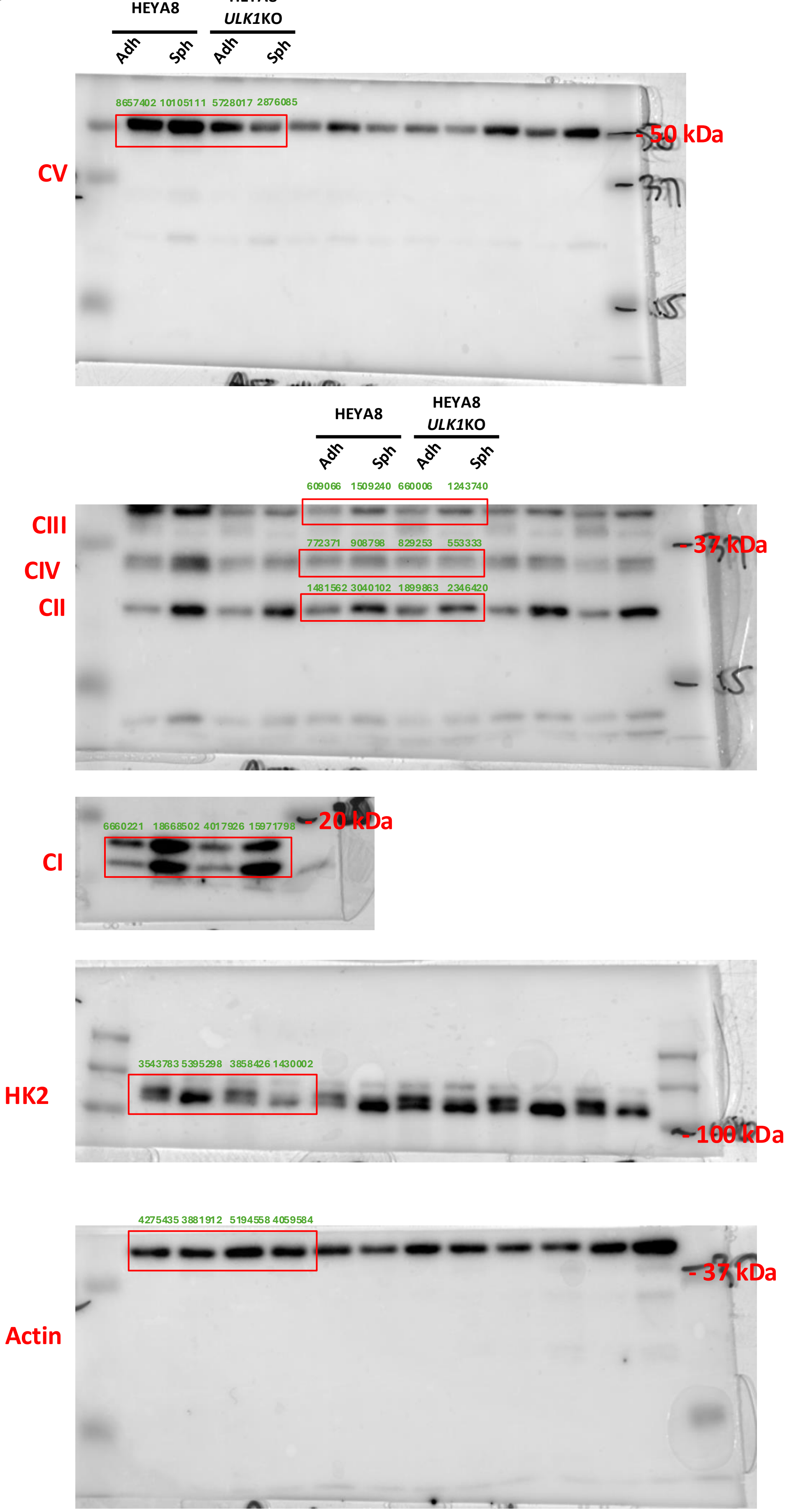

HEYA8
HEYA8 ULK1KO
Adh
Sph
Adh
Sph
8657402 10105111 5728017 2876085
50 kDa
CV
HEYA8
HEYA8 ULK1KO
Adh
Sph
Adh
Sph
609066 1509240 660006 1243740
CIII
772371 908798 829253 553333
CIV
37 kDa
1481562 3040102 1899863 2346420
CII
6660221 18668502 4017926 15971798
20 kDa
CI
3543783 5395298 3858426 1430002
HK2
100 kDa
4275435 3881912 5194558 4059584
37 kDa
Actin

Figure 4

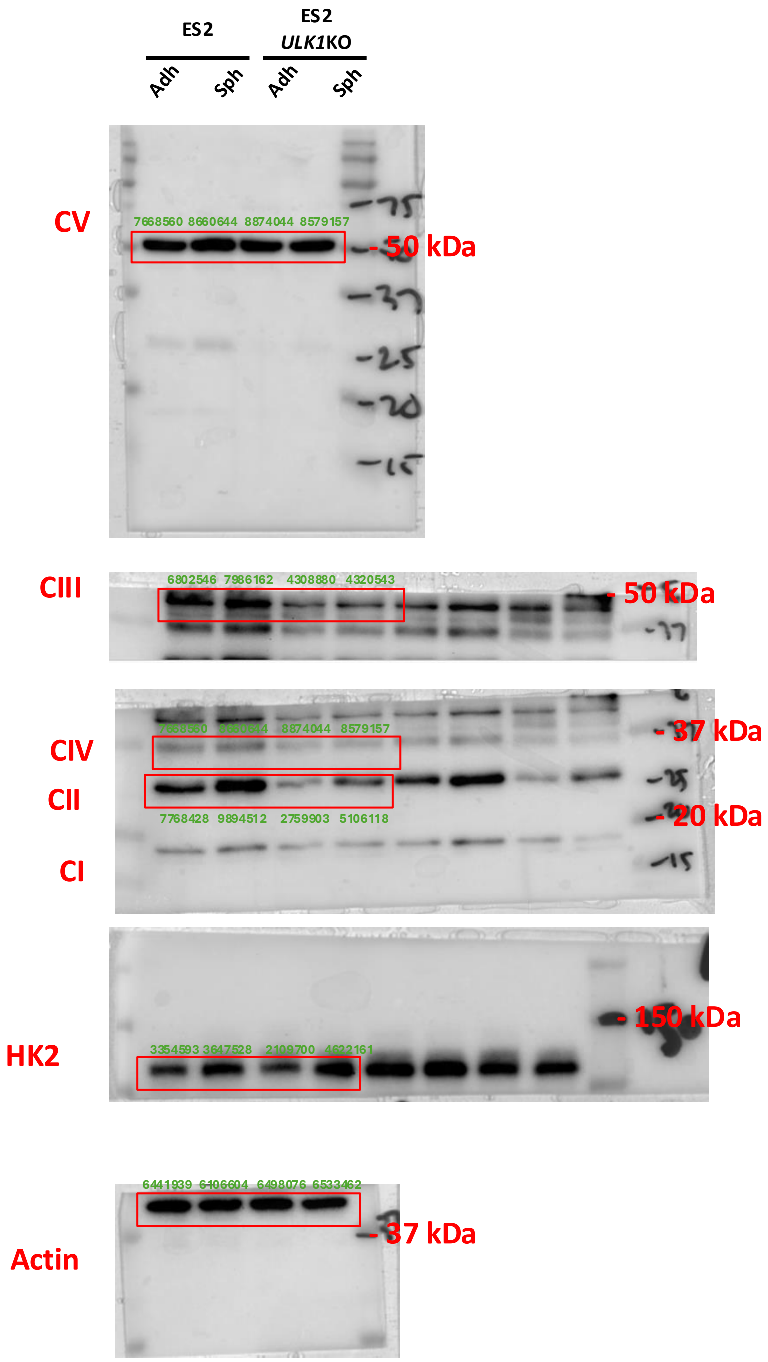

ES2
ES2 *ULK1*KO
Adh
Sph
Adh
Sph
CV
7668560 8660644 8874044 8579157
50 kDa
CIII
6802546 7986162 4308880 4320543
50 kDa
CIV
CII
CI
8874044 8579157
37 kDa
7768428 9894512 2759903 5106118
20 kDa
HK2
3354593 3647528 2109700 4622161
150 kDa
Actin
6441939 6406604 6498076 6533462
37 kDa

Supplementary Figure 1

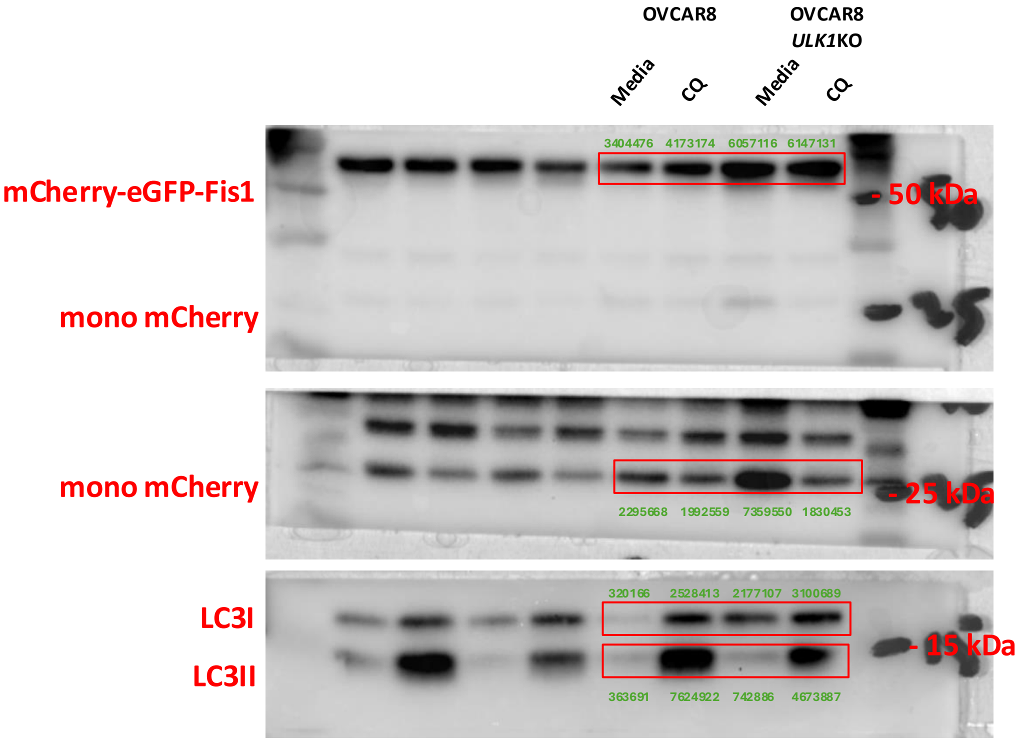

Supplementary Figure 2

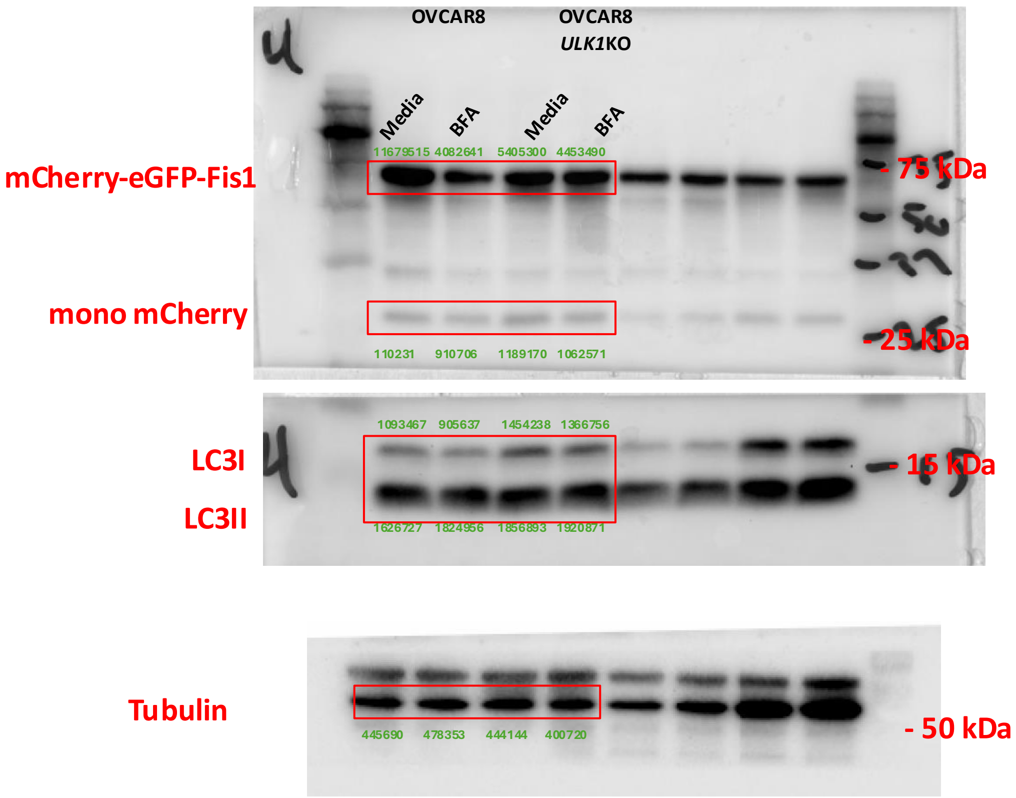

Supplementary Figure 3

A

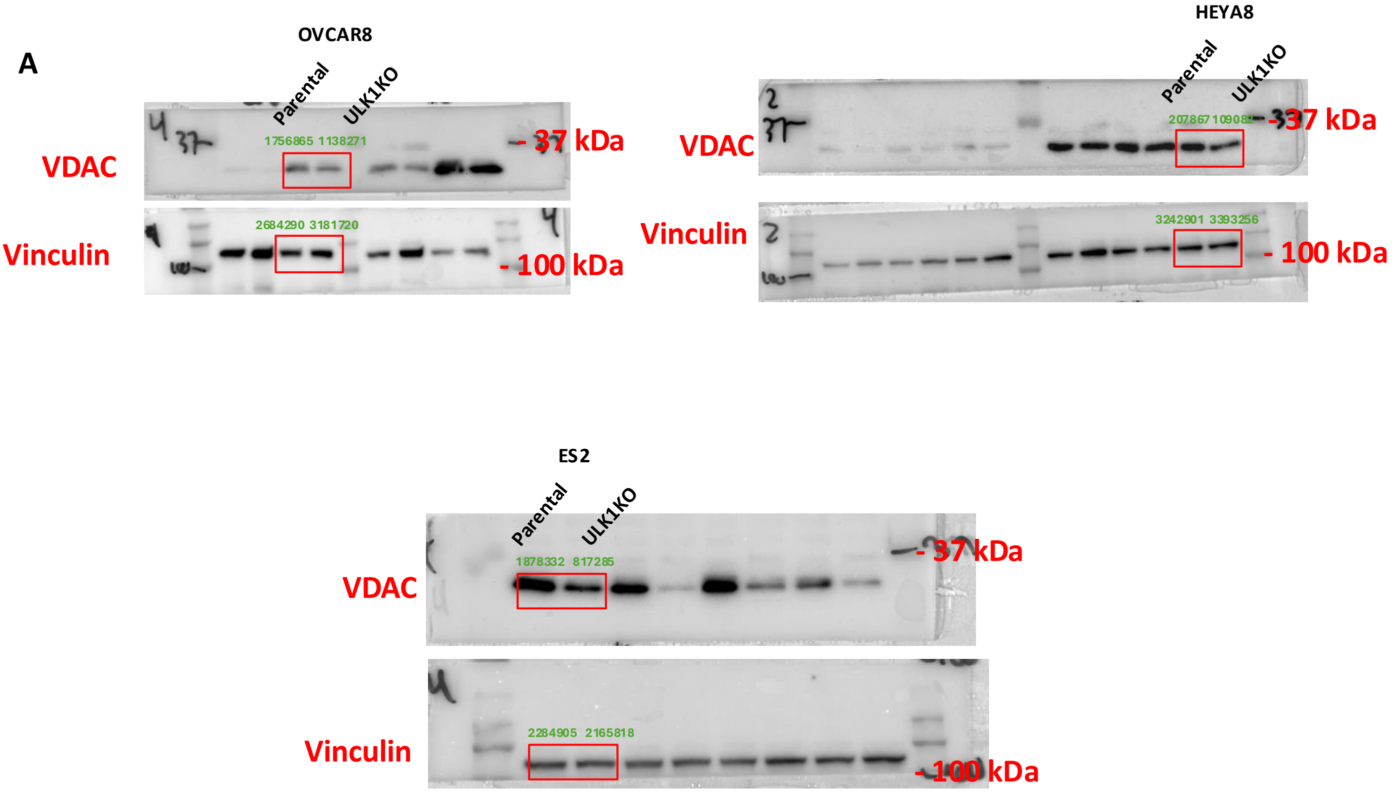

B

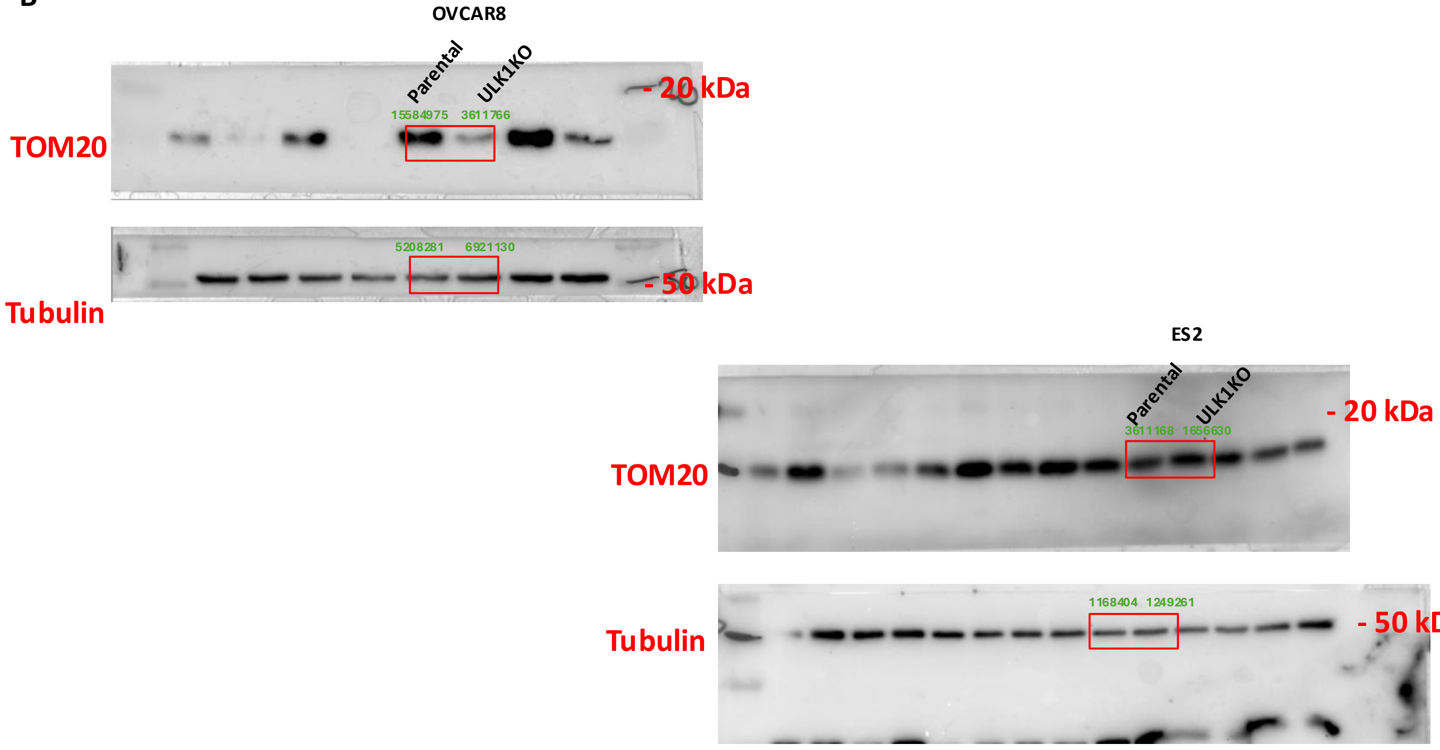

Supplementary Figure 4

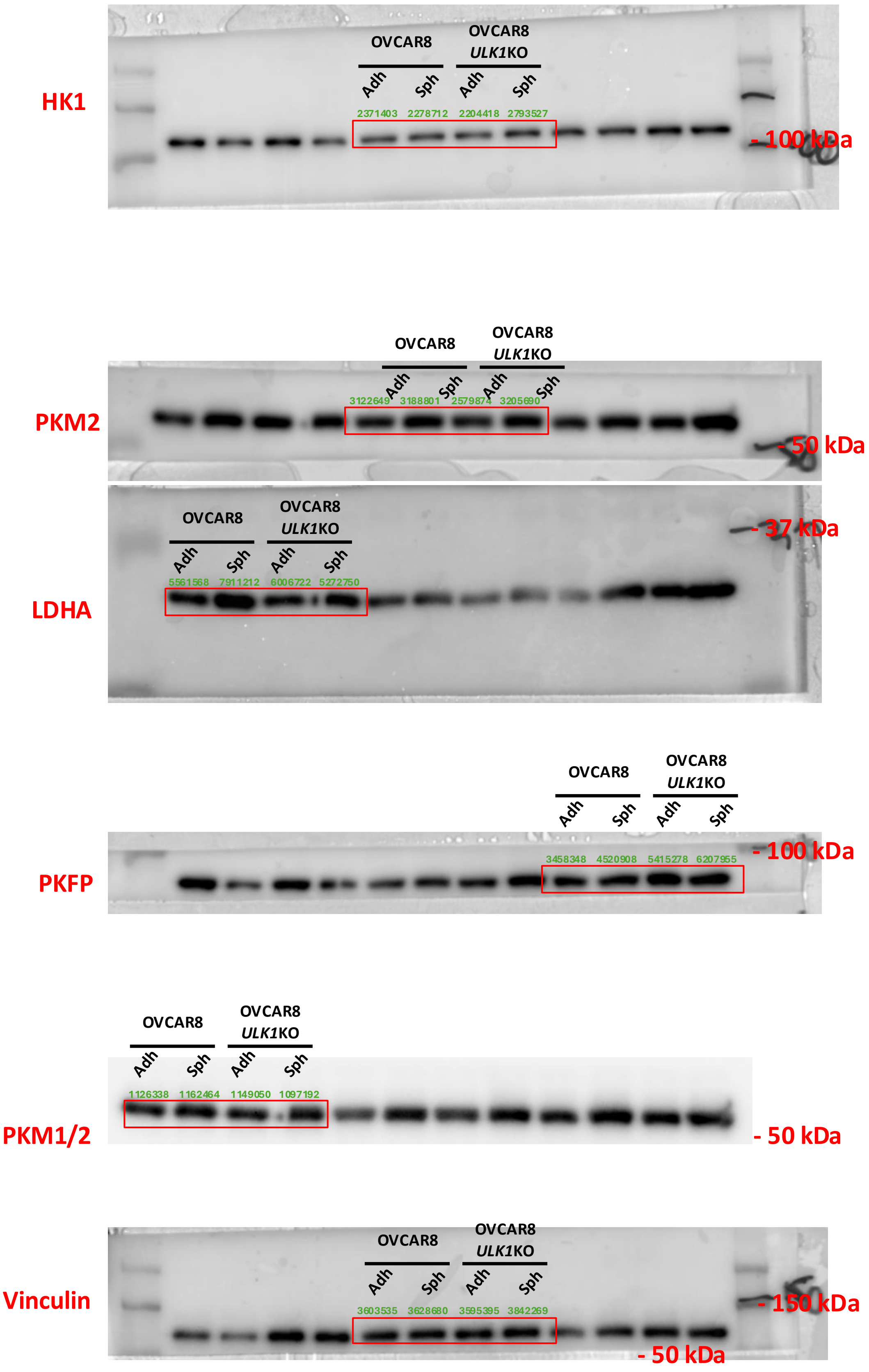

Supplementary Figure 4

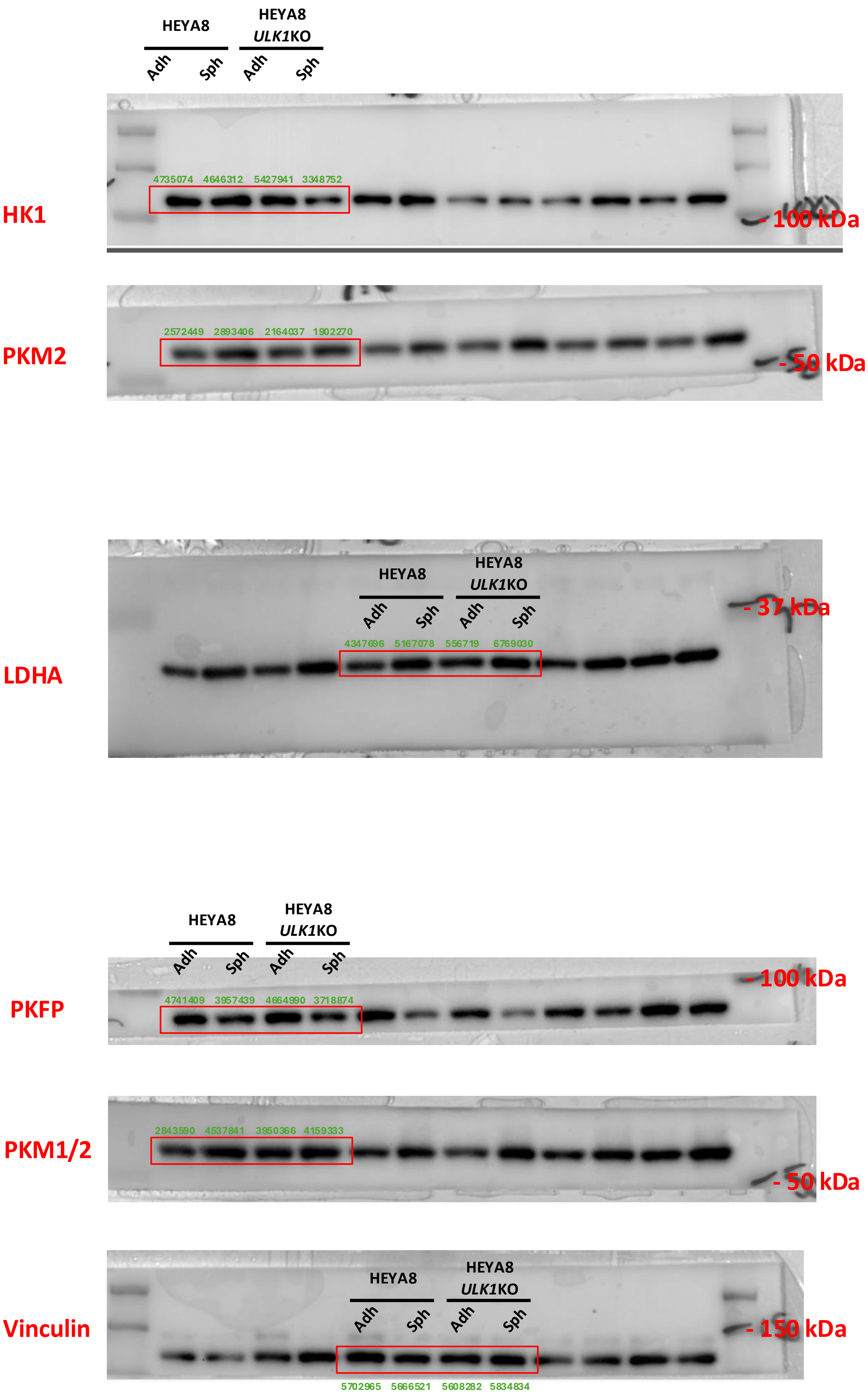

Supplement: Supplementary file 1 [file cancers-18-01746-s001.zip › cancers-4294561-File S1.pdf]
