# Supplementary material for: Divergent Role of ULK1 to Balance Mitochondrial Homeostasis and Bioenergetics in Ovarian Cancer Spheroids
Source: Cancers (Basel). 2026 May 27;18(11):1746. doi: 10.3390/cancers18111746 (PMC13255712; doi:10.3390/cancers18111746)
Supplement: Supplementary file 1 [file cancers-18-01746-s001.zip › Supplementary Figures.pdf]

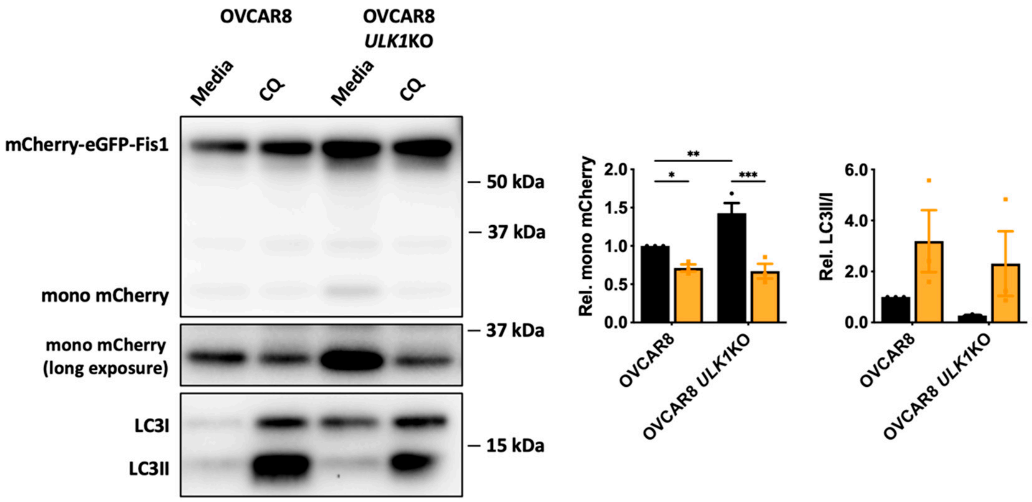

Supplementary Figure S1. Chloroquine treatment reveals lysosome-dependent regulation of mitoR processing in OVCAR8 parental and *ULK1*KO spheroids

OVCAR8 parental and *ULK1*KO-mitoR cells were seeded in spheroid culture for 24 h and treated with chloroquine (CQ; 50 µM) for an additional 24 h before collecting protein lysates. Densitometric analysis of mono-mCherry (mono-mCherry/(mono-mCherry+mCherry-eGFP-FIS1)) and LC3II:I relative to expression in parental adherent conditions. Data displayed as mean ± SEM; Two-way ANOVA followed by Šidák's multiple comparisons test, * $p < 0.05$, ** $p < 0.01$, *** $p < 0.001$.

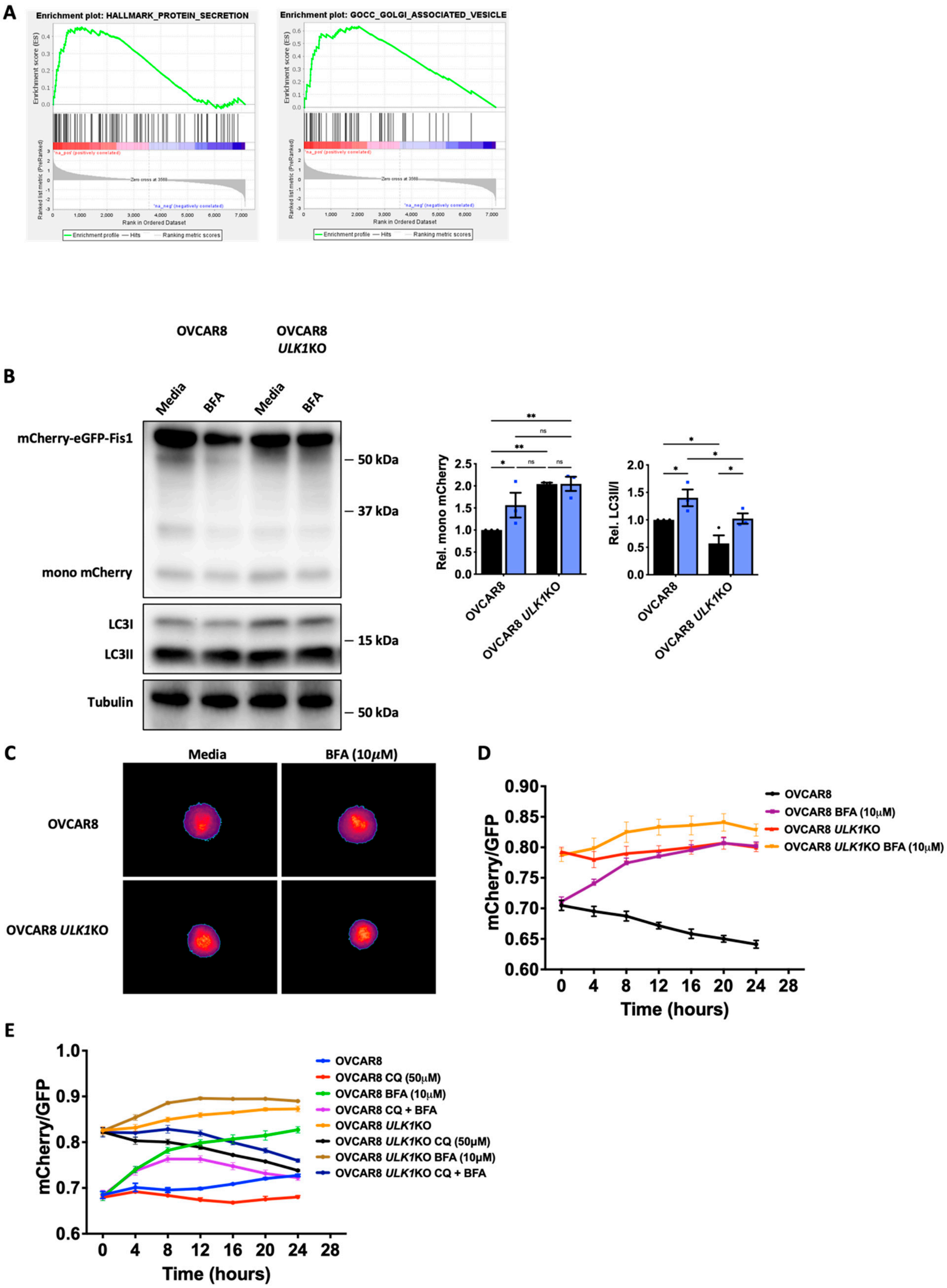
A
Enrichment plot: HALLMARK_PROTEIN_SECRETION
Enrichment plot: GOCC_GOLGI_ASSOCIATED_VESICLE
Enrichment score (ES)
Ranked list metric (PreRanked)
Rank in Ordered Dataset
Enrichment profile
Hits
Ranking metric scores
B
OVCAR8
OVCAR8 ULK1KO
Media
BFA
Media
BFA
mCherry-eGFP-Fis1
mono mCherry
LC3I
LC3II
Tubulin
50 kDa
37 kDa
15 kDa
50 kDa
Rel. mono mCherry
Rel. LC3II/I
OVCAR8
OVCAR8 ULK1KO
C
Media
BFA (10μM)
OVCAR8
OVCAR8 ULK1KO
D
mCherry/GFP
Time (hours)
OVCAR8
OVCAR8 BFA (10μM)
OVCAR8 ULK1KO
OVCAR8 ULK1KO BFA (10μM)
E
mCherry/GFP
Time (hours)
OVCAR8
OVCAR8 CQ (50μM)
OVCAR8 BFA (10μM)
OVCAR8 CQ + BFA
OVCAR8 ULK1KO
OVCAR8 ULK1KO CQ (50μM)
OVCAR8 ULK1KO BFA (10μM)
OVCAR8 ULK1KO CQ + BFA

Supplementary Figure S2. Brefeldin A enhances lysosome-associated mitochondrial reporter processing in OVCAR8 spheroids

A) Gene Set Enrichment Analysis of previously generated OVCAR8 and OVCAR8 *ULK1*KO spheroid proteomics showing enrichment of Hallmark Protein Secretion and Gene Ontology Cellular Component Golgi-Associated Vesicle signatures in OVCAR8 *ULK1*KO spheroids. B) OVCAR8 and OVCAR8 *ULK1*KO mitoR-expressing spheroids were cultured for 24 h and treated with brefeldin A (BFA; 10 µM) for an additional 24 h before protein lysates were collected. Densitometric analysis of mono-mCherry [mono-mCherry/ (mono-mCherry + mCherry-eGFP-FIS1)] and LC3II:I relative to OVCAR8 media-treated spheroids. Data are displayed as mean ± SEM; two-way ANOVA followed by Šidák's multiple comparisons test; N = 3 biological replicates. * $p < 0.05$, ** $p < 0.01$. C) Representative IncuCyte images of OVCAR8 and OVCAR8 *ULK1*KO mitoR-expressing spheroids treated with media control or BFA. D) Quantification of mitochondrial reporter activity as mCherry/eGFP ratio over time following BFA treatment (N = 3 biological replicates, with at least 6 technical replicates per biological replicate). E) Quantification of mCherry/eGFP ratio over time in OVCAR8 and OVCAR8 *ULK1*KO mitoR-expressing spheroids treated with media control, chloroquine (CQ; 50 µM), BFA (10 µM), or CQ + BFA (N = 3 biological replicates, with at least 6 technical replicates per biological replicate).

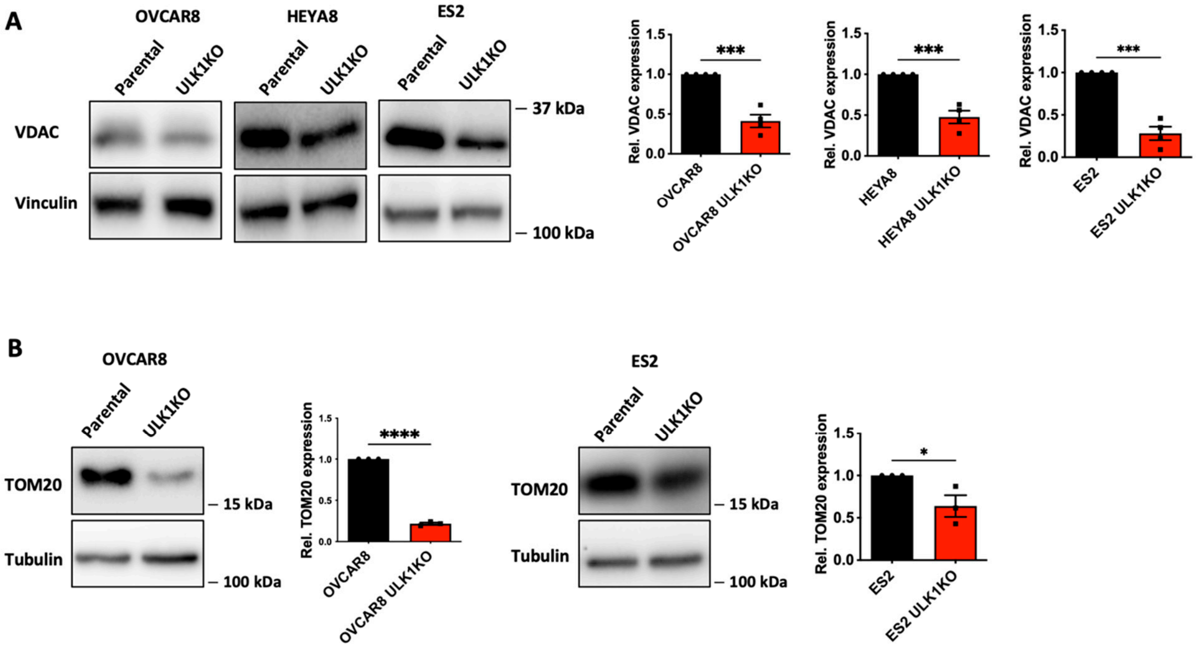

Supplementary Figure S3. ULK1 loss reduces mitochondrial protein abundance in EOC spheroids.

A) OVCAR8, HEYA8, and ES2 parental and *ULK1*KO cells were seeded in spheroid culture. Protein lysates were harvested 72 h after seeding for western blot analysis. Densitometric analysis of VDAC expression relative to parental spheroid conditions. B) OVCAR8 and ES2 parental and *ULK1*KO cells were seeded in spheroid culture. Protein lysates were harvested 72 h after seeding. Densitometric analysis of TOM20 expression relative to parental spheroid conditions. Data are displayed as mean ± SEM; Student's t-test; $N = 3–4$ biological replicates. * $p < 0.05$, *** $p < 0.001$, **** $p < 0.0001$.

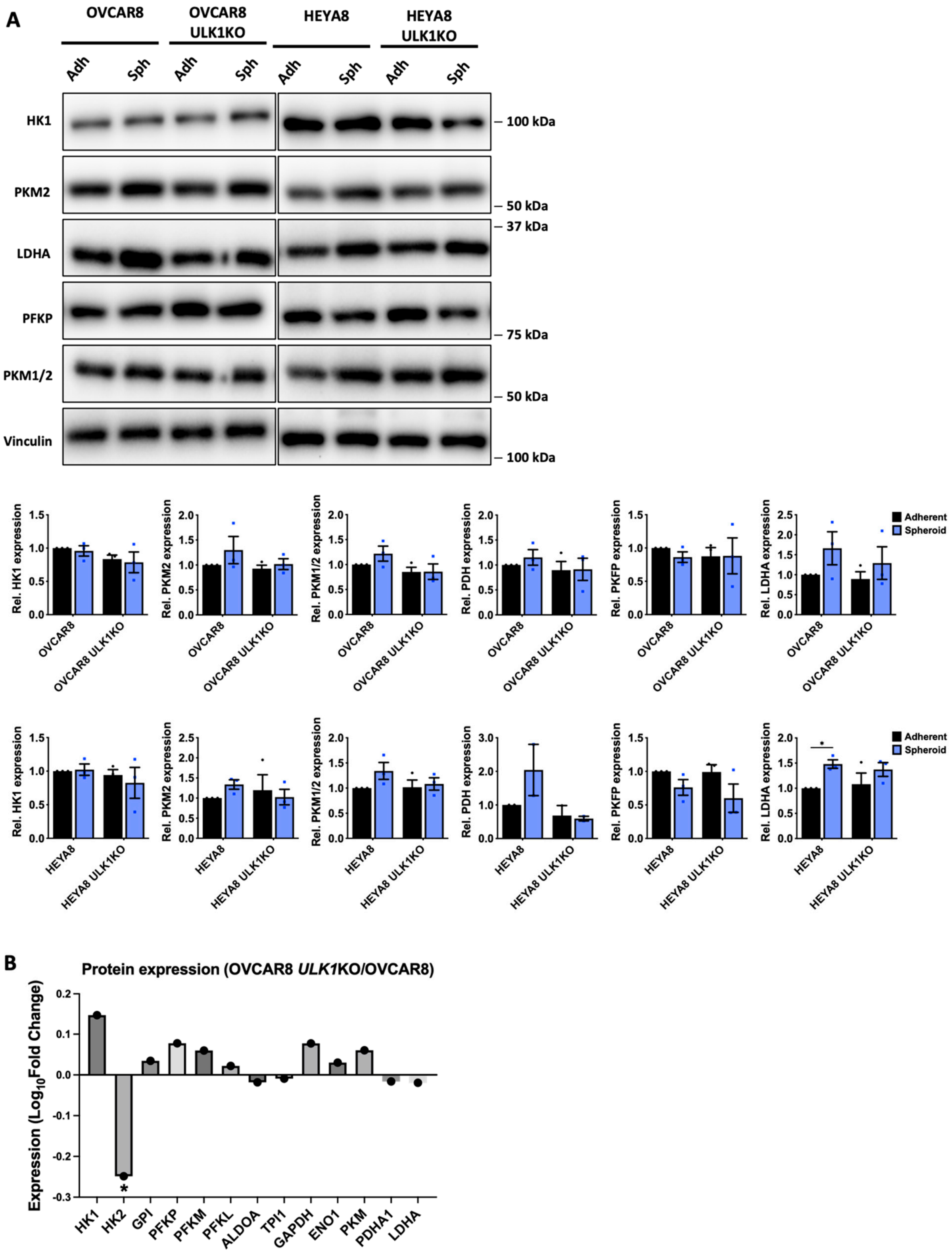
A
OVCAR8
OVCAR8 ULK1KO
HEYA8
HEYA8 ULK1KO
Adh
Sph
HK1
PKM2
LDHA
PFKP
PKM1/2
Vinculin
100 kDa
50 kDa
37 kDa
75 kDa
50 kDa
100 kDa
Rel. HK1 expression
Rel. PKM2 expression
Rel. PKM1/2 expression
Rel. PDH expression
Rel. PKFP expression
Rel. LDHA expression
Adherent
Spheroid
OVCAR8
OVCAR8 ULK1KO
HEYA8
HEYA8 ULK1KO
B
Protein expression (OVCAR8 ULK1KO/OVCAR8)
Expression (Log10Fold Change)
HK1
HK2
GPI
PFKP
PFKM
PFKL
ALDOA
TPI1
GAPDH
ENO1
PKM
PDHA1
LDHA

Supplementary Figure S4. Effects of *ULK1* loss on glycolysis protein abundance in EOC cells

A) OVCAR8, HEYA8, and ES2 parental and *ULK1*KO cells were seeded in adherent and spheroid culture. Protein lysates were harvested 72 h after seeding for western blot analysis for glycolysis proteins. Densitometric analysis of proteins relative to expression in parental adherent conditions. Data displayed as mean ± SEM; Two-way ANOVA followed by Šidák's multiple comparisons test, * $p < 0.05$. B) Expression of glycolysis proteins identified by label-free proteomics in OVCAR8 and OVCAR8 *ULK1*KO spheroids (*adj.P. < 0.05).
