# Supplementary material for: Divergent Role of ULK1 to Balance Mitochondrial Homeostasis and Bioenergetics in Ovarian Cancer Spheroids
Source: Cancers (Basel). 2026 May 27;18(11):1746. doi: 10.3390/cancers18111746 (PMC13255712; doi:10.3390/cancers18111746)
Supplement: Supplementary file 1 [file cancers-18-01746-s001.zip › Supplementary Tables.pdf]

Supplementary Table S1. ZIP synergy scores matrix for HEYA8 spheroids

Drug1	Drug2	Conc 1	Conc 2	Relative inhibition	Synergy	concUnit	SD	SEM
DCC-3116	METFORMIN	0	0	0	0	uM	0	0
DCC-3116	METFORMIN	0.16	0	-8.67	0	uM	20.4348306	11.7980549
DCC-3116	METFORMIN	0.31	0	1.79	0	uM	20.0896789	11.5987815
DCC-3116	METFORMIN	0.62	0	-1.12	0	uM	25.8095099	14.9011275
DCC-3116	METFORMIN	1.25	0	4.45	0	uM	21.8957256	12.6415031
DCC-3116	METFORMIN	2.5	0	-0.23	0	uM	23.6803484	13.6718555
DCC-3116	METFORMIN	5	0	3.65	0	uM	20.7440618	11.9765897
DCC-3116	METFORMIN	10	0	11.04	0	uM	16.5739323	9.5689643
DCC-3116	METFORMIN	20	0	22.38	0	uM	13.5406585	7.81770285
DCC-3116	METFORMIN	0	312.5	10.75	0	uM	5.83480362	3.36872544
DCC-3116	METFORMIN	0.16	312.5	10.92	-0.5705939	uM	23.3602661	13.4870559
DCC-3116	METFORMIN	0.31	312.5	14.34	0.20275141	uM	16.4194123	9.47975211
DCC-3116	METFORMIN	0.62	312.5	15.44	2.07659543	uM	15.6341197	9.02636324
DCC-3116	METFORMIN	1.25	312.5	16.42	2.35412664	uM	14.7364322	8.50808308
DCC-3116	METFORMIN	2.5	312.5	15.81	2.74868356	uM	8.32944976	4.80901006
DCC-3116	METFORMIN	5	312.5	21.91	7.33240452	uM	5.48965391	3.16945316
DCC-3116	METFORMIN	10	312.5	26.53	5.81918981	uM	13.3350303	7.69898334
DCC-3116	METFORMIN	20	312.5	35.21	7.78455157	uM	11.8364142	6.8337569
DCC-3116	METFORMIN	0	625	20.17	0	uM	8.11063705	4.68267848
DCC-3116	METFORMIN	0.16	625	16.02	0.69704619	uM	6.55187759	3.78272829
DCC-3116	METFORMIN	0.31	625	18.4	1.59406605	uM	3.75976506	2.17070137
DCC-3116	METFORMIN	0.62	625	25.2	3.53913848	uM	6.90236916	3.98508469
DCC-3116	METFORMIN	1.25	625	26.62	4.21342916	uM	12.9512406	7.47740225
DCC-3116	METFORMIN	2.5	625	24.26	5.40401624	uM	7.84793179	4.53100553
DCC-3116	METFORMIN	5	625	30.01	11.1929546	uM	6.34699141	3.6644372

DCC-3116	METFORMIN	10	625	34.88	8.70389495	uM	19.7505139	11.4029645
DCC-3116	METFORMIN	20	625	40.02	8.3125564	uM	13.6873774	7.90241102
DCC-3116	METFORMIN	0	1250	21.3	0	uM	2.20729095	1.27438002
DCC-3116	METFORMIN	0.16	1250	19.5	-2.1543722	uM	0.42	0.24248711
DCC-3116	METFORMIN	0.31	1250	17.95	-1.2400018	uM	4.78445748	2.76230781
DCC-3116	METFORMIN	0.62	1250	21.8	0.32795585	uM	1.19558912	0.6902737
DCC-3116	METFORMIN	1.25	1250	20.25	0.76239417	uM	4.05385413	2.34049378
DCC-3116	METFORMIN	2.5	1250	22.72	1.322115	uM	3.53010387	2.03810642
DCC-3116	METFORMIN	5	1250	27.22	5.60468267	uM	5.23402649	3.0218666
DCC-3116	METFORMIN	10	1250	31.32	3.3736438	uM	2.71545576	1.56776912
DCC-3116	METFORMIN	20	1250	41.64	6.67184695	uM	5.31006591	3.06576798
DCC-3116	METFORMIN	0	2500	25.26	0	uM	6.78933723	3.91982568
DCC-3116	METFORMIN	0.16	2500	12.77	-3.7678159	uM	5.36136488	3.09538545
DCC-3116	METFORMIN	0.31	2500	12.62	-2.7491402	uM	7.0267655	4.05690495
DCC-3116	METFORMIN	0.62	2500	11.93	-1.8109981	uM	3.90097424	2.25222853
DCC-3116	METFORMIN	1.25	2500	12.45	-1.3167279	uM	5.09222937	2.94
DCC-3116	METFORMIN	2.5	2500	14.36	-0.9611447	uM	4.1888463	2.41843154
DCC-3116	METFORMIN	5	2500	23.53	1.73527069	uM	7.77159143	4.4869304
DCC-3116	METFORMIN	10	2500	29.42	-5.1326091	uM	5.27401492	3.04495393
DCC-3116	METFORMIN	20	2500	38.09	0.63193627	uM	4.34362752	2.50779452
DCC-3116	METFORMIN	0	5000	26.77	0	uM	5.83267806	3.36749825
DCC-3116	METFORMIN	0.16	5000	22.6	-3.232488	uM	9.89861775	5.71496962
DCC-3116	METFORMIN	0.31	5000	28.08	-2.252389	uM	15.760295	9.09921059

DCC-3116	METFORMIN	0.62	5000	25.81	-2.5228611	uM	15.1689024	8.75776988
DCC-3116	METFORMIN	1.25	5000	28.98	-1.6565925	uM	11.947347	6.897804
DCC-3116	METFORMIN	2.5	5000	29.95	-1.1963622	uM	14.8344599	8.56467941
DCC-3116	METFORMIN	5	5000	29.73	0.73869055	uM	12.2043763	7.04619992
DCC-3116	METFORMIN	10	5000	38.19	-2.5854016	uM	7.28129338	4.20385669
DCC-3116	METFORMIN	20	5000	40.39	-2.812748	uM	9.59885583	5.541902
DCC-3116	METFORMIN	0	10000	35.6	0	uM	5.16574615	2.98244493
DCC-3116	METFORMIN	0.16	10000	35.94	-0.0335736	uM	13.17164	7.60464989
DCC-3116	METFORMIN	0.31	10000	34.42	0.3436146	uM	13.5901079	7.84625247
DCC-3116	METFORMIN	0.62	10000	34.41	-0.5690598	uM	14.0635143	8.11957374
DCC-3116	METFORMIN	1.25	10000	35.82	0.94265318	uM	6.76243546	3.90429393
DCC-3116	METFORMIN	2.5	10000	36.39	1.64524075	uM	8.50176452	4.90849604
DCC-3116	METFORMIN	5	10000	39.72	4.08678831	uM	8.17209276	4.71815995
DCC-3116	METFORMIN	10	10000	39.79	-3.5287708	uM	6.98857162	4.03485371
DCC-3116	METFORMIN	20	10000	39.11	-6.0417829	uM	8.85067417	5.10993912
DCC-3116	METFORMIN	0	20000	31.15	0	uM	9.04572275	5.22255046
DCC-3116	METFORMIN	0.16	20000	34.36	0.50135541	uM	7.96576634	4.59903734
DCC-3116	METFORMIN	0.31	20000	34.81	0.47325256	uM	5.11480205	2.95303234
DCC-3116	METFORMIN	0.62	20000	32.92	-0.0009471	uM	7.1109704	4.10552067
DCC-3116	METFORMIN	1.25	20000	36.84	1.18115851	uM	6.88736766	3.97642357
DCC-3116	METFORMIN	2.5	20000	32.3	0.66544139	uM	7.40072294	4.27280938
DCC-3116	METFORMIN	5	20000	38.25	2.57005903	uM	6.69219197	3.86373883

DCC-3116	METFORMIN	10	20000	36.21	-2.9008204	uM	9.1435788	5.27904768
DCC-3116	METFORMIN	20	20000	39.27	-6.9319515	uM	7.60938894	4.39328275
DCC-3116	METFORMIN	0	40000	36.49	0	uM	13.1628657	7.59958405
DCC-3116	METFORMIN	0.16	40000	32.14	0.22433674	uM	5.0229208	2.89998467
DCC-3116	METFORMIN	0.31	40000	32.28	0.17231489	uM	4.77577568	2.75729537
DCC-3116	METFORMIN	0.62	40000	32.3	-0.1961834	uM	4.81081074	2.77752288
DCC-3116	METFORMIN	1.25	40000	32.74	0.90008805	uM	4.73673235	2.7347537
DCC-3116	METFORMIN	2.5	40000	33.78	0.20606498	uM	5.05318052	2.91745513
DCC-3116	METFORMIN	5	40000	34.94	1.95369431	uM	4.91898702	2.83997848
DCC-3116	METFORMIN	10	40000	36.47	-0.4669224	uM	5.02241974	2.89969539
DCC-3116	METFORMIN	20	40000	39.65	-6.6760246	uM	3.2657975	1.88550907

Supplementary Table S2. ZIP synergy scores matrix for ES2 spheroids

Drug1	Drug2	Conc 1	Conc 2	Relative inhibition	Synergy	concUnit	SD	SEM
DCC-3116	METFORMIN	5	1250	34.97	11.3076391	uM	5.29537849	3.0572882
DCC-3116	METFORMIN	10	625	30.78	10.0632817	uM	6.77443233	3.91122033
DCC-3116	METFORMIN	10	1250	43.74	9.98977626	uM	14.7865119	8.53699661
DCC-3116	METFORMIN	1.25	40000	68.14	9.68025384	uM	3.00434685	1.73456046
DCC-3116	METFORMIN	5	625	25.42	9.60452758	uM	5.13553632	2.96500328
DCC-3116	METFORMIN	2.5	20000	53.42	9.45885501	uM	5.93520851	3.42669423
DCC-3116	METFORMIN	0.31	10000	43.39	9.41029255	uM	11.0134887	6.35864067
DCC-3116	METFORMIN	1.25	1250	29.47	8.75068018	uM	4.14212908	2.39145934
DCC-3116	METFORMIN	10	312.5	23.65	8.38583862	uM	3.42697145	1.97856289
DCC-3116	METFORMIN	0.16	40000	67.7	8.38204043	uM	2.69214289	1.55430942
DCC-3116	METFORMIN	5	20000	54.87	8.36007338	uM	5.48407087	3.16622979
DCC-3116	METFORMIN	0.62	20000	52.12	8.16576007	uM	8.071396	4.66002265
DCC-3116	METFORMIN	0.31	20000	51.08	7.83989991	uM	6.36167693	3.67291589
DCC-3116	METFORMIN	5	40000	68.43	7.75330231	uM	3.79073871	2.18858402
DCC-3116	METFORMIN	0.62	40000	67.97	7.64739439	uM	2.67561208	1.54476535
DCC-3116	METFORMIN	5	312.5	17.28	7.54252005	uM	2.36247751	1.36397703
DCC-3116	METFORMIN	2.5	1250	25.99	7.53604141	uM	11.1114311	6.41518771
DCC-3116	METFORMIN	1.25	312.5	16.69	7.21601863	uM	6.30354133	3.63935129
DCC-3116	METFORMIN	2.5	10000	44.78	7.08753977	uM	9.11030918	5.25983946
DCC-3116	METFORMIN	0.62	1250	25.97	6.83646754	uM	14.6520863	8.45938598
DCC-3116	METFORMIN	2.5	40000	68.29	6.60921474	uM	3.24623372	1.87421391
DCC-3116	METFORMIN	1.25	2500	32.44	6.56180979	uM	10.4750672	6.04778288
DCC-3116	METFORMIN	0.31	5000	39.73	6.52316563	uM	9.89868173	5.71500656
DCC-3116	METFORMIN	10	40000	69.38	6.31351649	uM	4.26373467	2.46166836
DCC-3116	METFORMIN	5	2500	30.98	5.89318128	uM	2.48861407	1.436802

DCC-3116	METFORMIN	0.31	2500	31.63	5.80652797	uM	3.92389263	2.26546046
DCC-3116	METFORMIN	0.62	2500	35.96	5.76736585	uM	7.48161302	4.31951129
DCC-3116	METFORMIN	0.31	1250	25.49	5.66164682	uM	9.9366594	5.73693298
DCC-3116	METFORMIN	0.16	1250	24.16	5.64175301	uM	32.5207324	18.7758536
DCC-3116	METFORMIN	0.62	312.5	9.53	5.64138281	uM	8.4730455	4.8919151
DCC-3116	METFORMIN	1.25	625	20.66	5.56744872	uM	7.04631346	4.06819097
DCC-3116	METFORMIN	1.25	20000	51.5	5.46095452	uM	6.18586561	3.57141117
DCC-3116	METFORMIN	0.16	20000	47.01	5.27455459	uM	5.99815805	3.46303817
DCC-3116	METFORMIN	5	10000	46.2	5.25907501	uM	7.66543758	4.42564245
DCC-3116	METFORMIN	10	20000	56.04	5.2372004	uM	5.00010333	2.886811
DCC-3116	METFORMIN	0.62	10000	41.63	5.21999408	uM	9.35606933	5.40172915
DCC-3116	METFORMIN	0.16	312.5	5.86	5.13760502	uM	7.90469059	4.56377524
DCC-3116	METFORMIN	5	5000	37.4	4.7689868	uM	8.94813016	5.16620536
DCC-3116	METFORMIN	2.5	2500	24.83	4.7110212	uM	4.301399	2.48341387
DCC-3116	METFORMIN	0.31	40000	67.96	4.69798603	uM	2.48083319	1.43230971
DCC-3116	METFORMIN	0.16	2500	30.85	4.66887918	uM	4.24649267	2.45171369
DCC-3116	METFORMIN	2.5	312.5	12.62	4.42512202	uM	4.94271518	2.85367794
DCC-3116	METFORMIN	2.5	5000	37.35	4.40492427	uM	9.62403761	5.55644071
DCC-3116	METFORMIN	0.62	5000	36.26	4.36849388	uM	11.0800376	6.39706269
DCC-3116	METFORMIN	0.31	312.5	9.96	4.25056793	uM	5.20790105	3.00678307
DCC-3116	METFORMIN	1.25	5000	29.92	4.17808926	uM	10.831985	6.25384948
DCC-3116	METFORMIN	0.62	625	11.52	4.09741762	uM	10.5346871	6.08220446
DCC-3116	METFORMIN	20	40000	72.27	3.79588854	uM	5.41467451	3.12616378
DCC-3116	METFORMIN	0.16	625	10.65	3.5458658	uM	11.1009654	6.40914538
DCC-3116	METFORMIN	10	2500	34.13	3.44977966	uM	8.76145536	5.05842861
DCC-3116	METFORMIN	2.5	625	14.6	3.17348597	uM	8.37205072	4.83360574
DCC-3116	METFORMIN	1.25	10000	38.64	2.88344445	uM	9.18163384	5.30101877

DCC-3116	METFORMIN	0.31	625	10.66	2.88315016	uM	7.33523915	4.2350023
DCC-3116	METFORMIN	10	10000	46.39	2.87784365	uM	8.57183955	4.94895387
DCC-3116	METFORMIN	20	20000	59.19	2.65048766	uM	6.43308117	3.71414114
DCC-3116	METFORMIN	10	5000	40.93	2.62338886	uM	9.04336774	5.2211908
DCC-3116	METFORMIN	0.16	5000	35.39	2.38171318	uM	10.3578376	5.98010033
DCC-3116	METFORMIN	20	1250	34.62	1.9288948	uM	9.91028254	5.72170429
DCC-3116	METFORMIN	0.16	10000	38.78	1.73084716	uM	9.20565587	5.31488789
DCC-3116	METFORMIN	20	625	34.44	1.34918883	uM	6.73594091	3.8889973
DCC-3116	METFORMIN	20	312.5	27.3	0.31249626	uM	4.89629452	2.82687696
DCC-3116	METFORMIN	0	0	0.1	0	uM	0	0
DCC-3116	METFORMIN	0.16	0	-0.37	0	uM	3.72825697	2.15251016
DCC-3116	METFORMIN	0.31	0	4.97	0	uM	1.28032548	0.73919626
DCC-3116	METFORMIN	0.62	0	5.17	0	uM	2.22919268	1.28702499
DCC-3116	METFORMIN	1.25	0	4.33	0	uM	3.65116876	2.10800327
DCC-3116	METFORMIN	2.5	0	9.37	0	uM	3.3883526	1.95626628
DCC-3116	METFORMIN	5	0	10.39	0	uM	3.21802735	1.85792895
DCC-3116	METFORMIN	10	0	17.88	0	uM	5.55706157	3.208371
DCC-3116	METFORMIN	20	0	27.18	0	uM	9.01483777	5.20471901
DCC-3116	METFORMIN	0	312.5	3.04	0	uM	2.17111339	1.2534929
DCC-3116	METFORMIN	0	625	12.94	0	uM	1.04892008	0.60559429
DCC-3116	METFORMIN	0	1250	21.31	0	uM	1.37201798	0.79213495
DCC-3116	METFORMIN	0	2500	27.14	0	uM	8.03720723	4.64028376
DCC-3116	METFORMIN	0	5000	27.5	0	uM	9.69661281	5.59834202
DCC-3116	METFORMIN	0	10000	33.85	0	uM	9.80482194	5.66081659
DCC-3116	METFORMIN	0	20000	45.07	0	uM	6.64930322	3.838977
DCC-3116	METFORMIN	0	40000	60.6	0	uM	4.28327367	2.47294921
DCC-3116	METFORMIN	20	10000	51.4	-0.9235046	uM	6.58045844	3.79922945

DCC-3116	METFORMIN	20	2500	37.49	-2.5105695	uM	10.4976013	6.06079294
DCC-3116	METFORMIN	20	5000	43.53	-2.5310101	uM	7.41603892	4.28165207

Supplementary Table S3. ZIP synergy scores matrix for OVCAR8 spheroids

Drug1	Drug2	Conc1	Conc 2	Relative inhibition	Synergy	concUnit	SD	SEM
DCC-3116	METFORMIN	20	312.5	12.79	4.56057179	uM	22.62424	13.062111
DCC-3116	METFORMIN	0.31	1250	36.73	3.07714116	uM	15.4341839	8.91093024
DCC-3116	METFORMIN	20	625	26.57	2.79971842	uM	20.1713683	11.6459449
DCC-3116	METFORMIN	2.5	312.5	2.37	2.26130524	uM	25.5313343	14.7405228
DCC-3116	METFORMIN	20	40000	63.92	1.39946145	uM	4.40105669	2.54095127
DCC-3116	METFORMIN	0.62	312.5	-2.24	1.03812806	uM	26.8656212	15.5108736
DCC-3116	METFORMIN	10	312.5	2.27	0.69559036	uM	23.405515	13.5131804
DCC-3116	METFORMIN	2.5	625	24.76	0.38803478	uM	16.6100492	9.58981636
DCC-3116	METFORMIN	0	0	0.1	0	uM	0	0
DCC-3116	METFORMIN	0.16	0	-27.7	0	uM	35.0173476	20.2172751
DCC-3116	METFORMIN	0.31	0	-29.46	0	uM	42.245356	24.3903676
DCC-3116	METFORMIN	0.62	0	-28.11	0	uM	39.1197601	22.585804
DCC-3116	METFORMIN	1.25	0	-24.16	0	uM	34.9024775	20.1509548
DCC-3116	METFORMIN	2.5	0	-24.39	0	uM	36.033236	20.8037985
DCC-3116	METFORMIN	5	0	-25.36	0	uM	37.9652029	21.9192201
DCC-3116	METFORMIN	10	0	-32.18	0	uM	37.9365839	21.9026969
DCC-3116	METFORMIN	20	0	-33.62	0	uM	36.9184471	21.3148753
DCC-3116	METFORMIN	0	312.5	7.19	0	uM	27.9211389	16.1202771
DCC-3116	METFORMIN	0	625	24.49	0	uM	19.5761624	11.3023026
DCC-3116	METFORMIN	0	1250	37.01	0	uM	14.3904378	8.30832314
DCC-3116	METFORMIN	0	2500	43.77	0	uM	10.9555511	6.32519038
DCC-3116	METFORMIN	0	5000	52.6	0	uM	10.7700015	6.21806329
DCC-3116	METFORMIN	0	10000	59.26	0	uM	10.6300392	6.13725599
DCC-3116	METFORMIN	0	20000	60.56	0	uM	10.2927223	5.94250602
DCC-3116	METFORMIN	0	40000	58.41	0	uM	8.94680017	5.16543749

DCC-3116	METFORMIN	0.31	625	25.91	-0.0748652	uM	17.5274252	10.1194636
DCC-3116	METFORMIN	0.62	625	26.99	-0.1562312	uM	22.2222209	12.8300052
DCC-3116	METFORMIN	0.16	312.5	-4.55	-0.2997304	uM	30.0718628	17.3619981
DCC-3116	METFORMIN	10	40000	60.77	-0.5320306	uM	5.38376572	3.10831859
DCC-3116	METFORMIN	5	312.5	-4.16	-0.6484502	uM	25.6121657	14.7871908
DCC-3116	METFORMIN	10	625	19.21	-0.9486807	uM	17.4575409	10.0791159
DCC-3116	METFORMIN	1.25	312.5	-2.97	-0.9965501	uM	29.249253	16.8870641
DCC-3116	METFORMIN	0.16	40000	56.37	-1.264028	uM	6.86657848	3.96442093
DCC-3116	METFORMIN	5	40000	59.38	-1.4674771	uM	5.97226088	3.44808643
DCC-3116	METFORMIN	0.16	625	22.39	-1.7140755	uM	23.4202099	13.5216645
DCC-3116	METFORMIN	2.5	40000	57.52	-1.7323129	uM	6.35302553	3.667921
DCC-3116	METFORMIN	0.62	40000	56.27	-1.7408254	uM	6.70898154	3.8734323
DCC-3116	METFORMIN	1.25	625	20.89	-1.7691887	uM	15.4480236	8.91892059
DCC-3116	METFORMIN	5	625	20.06	-2.0605659	uM	19.5409681	11.2819832
DCC-3116	METFORMIN	1.25	40000	57.11	-2.2446372	uM	6.60144176	3.81134418
DCC-3116	METFORMIN	0.16	10000	54.38	-2.3890697	uM	7.34234976	4.23910761
DCC-3116	METFORMIN	0.16	20000	57.27	-2.7562591	uM	7.93206362	4.57957907
DCC-3116	METFORMIN	20	20000	46.12	-2.9188198	uM	9.32087085	5.38140729
DCC-3116	METFORMIN	0.31	312.5	-3.09	-2.9785628	uM	31.0780324	17.9429104
DCC-3116	METFORMIN	5	20000	53.82	-3.0926848	uM	8.16515156	4.71415245
DCC-3116	METFORMIN	0.62	20000	57.79	-3.2358519	uM	8.42147849	4.86214287
DCC-3116	METFORMIN	0.31	40000	56.33	-3.2504187	uM	6.79674432	3.92410216
DCC-3116	METFORMIN	2.5	20000	54.73	-3.2579818	uM	7.04954136	4.0700546
DCC-3116	METFORMIN	10	20000	51.01	-3.3301212	uM	8.98525459	5.18763915
DCC-3116	METFORMIN	0.62	10000	54.29	-3.5966184	uM	7.12762466	4.11513602
DCC-3116	METFORMIN	1.25	20000	56.65	-3.7449666	uM	6.97244816	4.02554482
DCC-3116	METFORMIN	0.62	1250	35.05	-3.9437729	uM	21.2775617	12.284606

DCC-3116	METFORMIN	0.31	10000	55.63	-4.5418568	uM	6.59582696	3.80810247
DCC-3116	METFORMIN	1.25	1250	31.21	-4.6934121	uM	17.6284581	10.177795
DCC-3116	METFORMIN	0.31	20000	59.25	-4.743432	uM	6.64951878	3.83910146
DCC-3116	METFORMIN	1.25	10000	51.23	-5.040598	uM	6.10051091	3.52213161
DCC-3116	METFORMIN	0.31	2500	36.76	-5.0433504	uM	14.6422232	8.4536915
DCC-3116	METFORMIN	5	10000	52.4	-5.2207271	uM	7.94592348	4.58758106
DCC-3116	METFORMIN	0.16	1250	30.66	-5.7353793	uM	20.2768571	11.7068489
DCC-3116	METFORMIN	2.5	10000	49.53	-5.7558723	uM	8.37756528	4.83678957
DCC-3116	METFORMIN	2.5	1250	31.91	-5.8221233	uM	17.1370135	9.89405938
DCC-3116	METFORMIN	10	10000	49.23	-5.8666958	uM	7.23775057	4.17871724
DCC-3116	METFORMIN	20	1250	29.65	-6.3756353	uM	17.8831606	10.3248476
DCC-3116	METFORMIN	20	10000	48.76	-7.028358	uM	8.12251398	4.68953563
DCC-3116	METFORMIN	0.62	5000	44.06	-7.0421661	uM	7.59954165	4.38759742
DCC-3116	METFORMIN	1.25	5000	46.54	-7.1045713	uM	9.15316521	5.2845824
DCC-3116	METFORMIN	0.31	5000	46.16	-7.1941769	uM	6.5549447	3.78449909
DCC-3116	METFORMIN	0.16	5000	46.9	-7.223596	uM	10.1417076	5.85531762
DCC-3116	METFORMIN	5	1250	27.95	-8.8419745	uM	17.5857167	10.1531183
DCC-3116	METFORMIN	10	1250	26.06	-8.8971296	uM	19.5075891	11.2627118
DCC-3116	METFORMIN	1.25	2500	35.34	-9.5507832	uM	13.4834009	7.78464514
DCC-3116	METFORMIN	5	5000	44.35	-9.6887316	uM	10.8643653	6.27254423
DCC-3116	METFORMIN	0.62	2500	34.57	-10.191448	uM	14.4873704	8.36428718
DCC-3116	METFORMIN	2.5	5000	40.12	-10.863311	uM	8.56493043	4.94496489
DCC-3116	METFORMIN	0.16	2500	30.94	-11.789244	uM	18.5096542	10.6865539
DCC-3116	METFORMIN	10	5000	40.29	-12.699315	uM	8.2552347	4.76616198
DCC-3116	METFORMIN	5	2500	30.79	-13.961968	uM	12.3873739	7.15185368
DCC-3116	METFORMIN	2.5	2500	32.49	-14.009199	uM	15.6782216	9.05182548
DCC-3116	METFORMIN	20	5000	39.43	-14.714836	uM	8.9006011	5.13876444

DCC-3116	METFORMIN	20	2500	32.18	-15.155552	uM	11.0682022	6.39022952
DCC-3116	METFORMIN	10	2500	30.73	-15.743731	uM	10.9721572	6.33477791
